# Drought susceptibility of modern rice varieties: an effect of linkage of drought tolerance with undesirable traits

**DOI:** 10.1038/srep14799

**Published:** 2015-10-13

**Authors:** Prashant Vikram, B. P. Mallikarjuna Swamy, Shalabh Dixit, Renu Singh, Bikram P. Singh, Berta Miro, Ajay Kohli, Amelia Henry, N. K. Singh, Arvind Kumar

**Affiliations:** 1 Plant Breeding, Genetics, and Biotechnology Division, International Rice Research Institute, Los Baños, Philippines; 2 National Research Center for Plant Biology, Indian Agricultural Research Institute, New Delhi, India 110012; 3 Crop and Environmental Sciences Division, International Rice Research Institute, Los Baños, Philippines

## Abstract

Green Revolution (GR) rice varieties are high yielding but typically drought sensitive. This is partly due to the tight linkage between the loci governing plant height and drought tolerance. This linkage is illustrated here through characterization of *qDTY*_*1.1*_, a QTL for grain yield under drought that co-segregates with the GR gene *sd1* for semi-dwarf plant height. We report that the loss of the *qDTY*_*1.1*_ allele during the GR was due to its tight linkage in repulsion with the *sd1* allele. Other drought-yield QTLs (*qDTY)* also showed tight linkage with traits rejected in GR varieties. Genetic diversity analysis for 11 different *qDTY* regions grouped GR varieties separately from traditional drought-tolerant varieties, and showed lower frequency of drought tolerance alleles. The increased understanding and breaking of the linkage between drought tolerance and undesirable traits has led to the development of high-yielding drought-tolerant dwarf lines with positive *qDTY* alleles and provides new hope for extending the benefits of the GR to drought-prone rice-growing regions.

The introduction of Green Revolution (GR) genes in rice and wheat is one of the most significant achievements in the history of agricultural research. The introduction of the dwarfing allele of the *sd1* gene led to the development of dwarf, high-yielding, lodging-resistant, fertilizer-responsive rice varieties that provided yield gains and food security in rice. Breeding efforts since the GR era have continued to incorporate the dwarf *sd1* allele, and traditional tall varieties adapted to rainfed conditions have consequently largely been replaced by dwarf GR rice varieties^1^ that show substantial yield gain in irrigated areas^2^. Undeniably, the GR has enabled us to improve the food security of rice consumers in past decades. The high yield potential and preferred grain quality of some of these GR varieties have led to their cultivation on large areas in rainfed regions^3^. However, rainfed rice-growing areas prone to abiotic stresses such as drought continue to have low productivity, and large yield losses are observed in GR varieties under drought because of their high sensitivity to drought^3^,^4^,^5^,^6^. Rainfed rice-growing areas comprise 45% of the total cultivated rice-growing area in the world, where drought in mild, moderate, or severe forms occurs in most years^6^. Given the large area under rainfed rice cultivation and the increasing incidence of drought stress in a range of cultivation systems because of receding water-table depths, improving the drought tolerance of GR varieties has a large potential for helping to achieve sustainable rice production. The annual yield gain of 1% for rice at the global level is far below the required 2% increase to meet rice demand in the coming decades^7^. Genetic improvement of rice for increased adaptation to rainfed environments can significantly help to fill this gap and improve food security in Asia and Africa.

To date, improvement of popular high-yielding GR varieties’ tolerance of abiotic stress has been achieved by crossing with traditional varieties or wild relatives^8^,^9^,^10^, for which the traditional varieties were used as donors and the high-yielding GR varieties provided the desired yield potential and preferred grain quality. Large segregating populations are needed in order to incorporate genes conferring tolerance of abiotic stresses in such crosses, which typically show poor recombination. One of the reasons for this could be the linkage/pleiotropic action of abiotic stress tolerance genes to traits that were considered undesirable, such as low yield potential, tall height, poor grain quality, etc., leading to their elimination from GR varieties during the selection process.

Because of the complexity of drought stress and the range of conditions to which rainfed rice crops are exposed, a multifaceted breeding strategy including marker-assisted approaches is necessary to improve drought tolerance in rice. In the past decade, the availability of molecular markers coupled with precision phenotyping has led to the identification of several quantitative trait loci (QTLs) for grain yield under drought (*qDTY*)^11^. Most of the *qDTY* were contributed by traditional varieties and are being deployed to develop drought-tolerant versions of popular GR varieties^12^. A major QTL, *qDTY*_*1.1*_, from the traditional variety N22 showed consistent effects in the backgrounds of popular GR varieties^5^,^13^. In addition to the drought tolerance allele of *qDTY*_*1.1*_, N22 possesses the tallness allele of the *sd1* gene for gibberellic acid synthesis (*OsGA20 oxidase*). The GR varieties IR64, Swarna, and MTU1010 possess the drought-susceptible allele of *qDTY*_*1.1*_ and the dwarfing allele of the *sd1* gene, which has a 383-bp deletion causing loss of function of *OsGA20 oxidase*^14^. The *qDTY*_*1.1*_ region harboring *sd1* showed a strong association with plant height, explaining around 50% of the phenotypic variation in two of the three mapping populations^5^. The *qDTY*_*1.1*_ effect on grain yield under drought was predicted to be either because of the pleiotropic effects of the *sd1* gene itself or due to a separate locus within the *qDTY*_*1.1*_ region tightly linked to *sd1*^15^,^16^,^17^,^18^. Both cases are a concern for breeding efforts to improve the drought tolerance of GR varieties, since linkage of *qDTY*_*1.1*_ and *sd1* would indicate that the drought tolerance allele could have been lost in developing dwarf varieties, and pleiotropy would indicate that drought susceptibility and dwarfness cannot be separated. Therefore, knowledge of the linkage or pleiotropic effect of *qDTY*_*1.1*_ and *sd1* will help in future strategizing for breeding drought-tolerant rice varieties.

The tallness and drought tolerance of *qDTY*_*1.1*_ lines and traditional rice varieties as compared with the dwarf plant height and drought susceptibility of GR varieties underline the importance of this locus for drought response. Similar to *qDTY*_*1.1*_, other *DTY* QTLs such as *qDTY*_*6.2*_*, qDTY*_*3.1*_, and *qDTY*_*3.2*_ also showed associations with tall plant height, reduced yield under irrigated conditions, and early days to flowering (DTF), respectively. The association of *qDTY* with such characteristics could be due to the close linkage of genes affecting these traits with genes for drought tolerance, or due to pleiotropic genes underlying these QTL regions. The rejection of these undesired traits during breeding since the GR era may have led to a loss of the positive *qDTY* alleles. We therefore hypothesized that the genomic alterations that occurred at the *qDTY*_*1.1*_ or *sd1* locus and other *qDTY* loci (due to selection) have contributed to the increased drought susceptibility of post-GR rice varieties. Characterization of genetic phenomena such as linkage and pleiotropic gene action in relation to stress response will contribute to understanding the genetic control of complex traits, which can facilitate more efficient crop improvement for stress tolerance.

To determine whether the GR selected against drought tolerance in rice, the objective of the present study was to characterize the drought tolerance QTL *qDTY*_*1.1*_ in relation to the GR dwarfness gene *sd1* by testing for linkage vs pleiotropy. The relationship between other *qDTY* with undesirable traits and the presence of *qDTY* in pre- and post-GR varieties was also investigated to determine the likelihood that *qDTY* were selected against because of their relationship with undesirable traits. Finally, we aimed to break the linkage between *qDTY*_*1.1*_ and the tall allele of *sd1,* as well as between other *qDTY* and undesirable traits, to develop high-yielding drought-tolerant rice varieties.

## Results

### Analysis of pleiotropy vs tight linkage between *sd1* and *qDTY*_*1.1*_

To determine whether the drought tolerance conferred by *qDTY*_*1.1*_ was due to a pleiotropic effect of the *sd1* gene or whether there was a tight genetic linkage between *qDTY*_*1.1*_ and *sd1* genes, a two-way approach was undertaken: (i) statistical analysis of multiple traits for linkage vs pleiotropy by saturation mapping in the recombinant inbred line (RIL) mapping populations used for the identification of *qDTY*_*1.1*_, and (ii) fine mapping of the *qDTY*_*1.1*_ locus using region-specific single nucleotide polymorphism (SNP) markers in a large backcross inbred line (BIL) population.

### Multiple trait analysis and re-mapping of sd1/qDTY_1.1_ in N22-derived RIL populations

**Figure 1:**
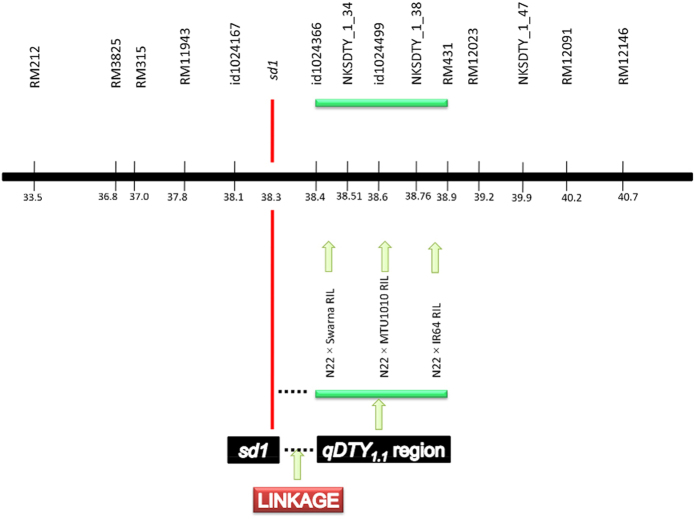
Improved resolution of *qDTY*_*1.1*_ in three different N22-derived RIL mapping populations (N22/Swarna, N22/IR64, and N22/MTU1010) showing the location of *qDTY*_*1.1*_ distal to the *sd1* gene between markers id1024366 (38.4 Mb) and RM431 (38.9 Mb). Positions of the markers are shown on the chromosome bar in Mbp.

Multiple trait analysis in N22/IR64 and N22/Swarna RIL populations did not show any significant pleiotropic effects of *qDTY*_*1.1*_ on DTF, plant height (PH), or harvest index (HI) under reproductive-stage drought stress (RS) (Table 1). A re-mapping of the *qDTY*_*1.1*_ QTL interval (RM11943–RM431) in the three N22-derived RIL populations (N22/Swarna, N22/IR64, and N22/MTU1010) using three additional SNP markers in the region revealed that *qDTY*_*1.1*_ was located distal to the *sd1* gene (Fig. 1, Table 2). The QTL intervals in the N22/Swarna, N22/IR64, and N22/MTU1010 populations were id1024366–id1024499, id1024499–RM431, and id1024366–id1024499, respectively. Further, *sd1* gene-based marker analysis of 161 RILs (48 from N22/Swarna, 57 from N22/IR64, and 56 from N22/ MTU1010) based on flanking markers of the *sd1* gene (Fig. 1) revealed the *qDTY*_*1.1*_ position to be below that of the *sd1* gene, between interval id1024366−id1024499 in a combined analysis over both years (Table 2).

### Fine mapping of the sd1/qDTY_1.1_ locus

**Figure 2:**
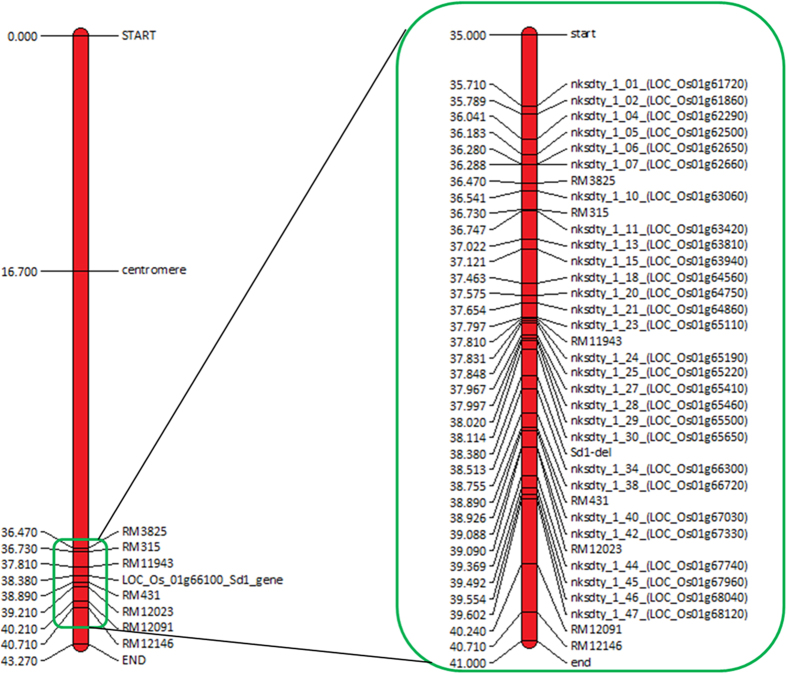
Map representing positions (Mbp) of the Sequenom MassARRAY custom SNP assay markers used for fine mapping of the *qDTY*_*1.1*_ QTL region.

**Figure 3:**
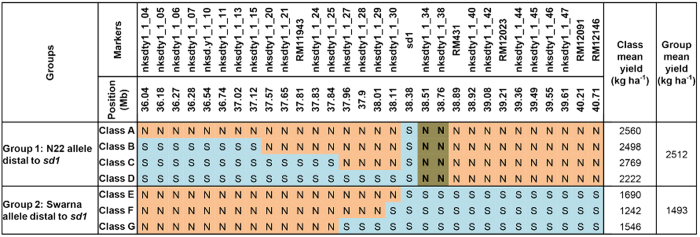
Recombination break point analysis of dwarf recombinant BILs possessing different combinations of N22 (N) and Swarna (S) alleles in the *qDTY*_*1.1*_ region and their effect on grain yield under drought. Of the seven classes presented in this figure, four (classes A, B, C, and D) showed N22 alleles distal to *sd1* and showed the highest mean yields under drought. Three classes (E, F, and G) had the Swarna allele distal to *sd1* and showed lower mean yields under drought than classes A-D. The numbers of individuals in classes A, B, C, D, E, F, and G were 3, 2, 2, 6, 2, 3, and 2, respectively. This suggests that markers nksdty1_1_34 and nksdty1_1_38 are most closely related to grain yield under drought.

In a recombination breakpoint analysis of the N22/2* Swarna BIL population with fixed DTF and *sd1* alleles that segregated for markers in the *qDTY*_*1.1*_ interval only (Fig. 2, Fig. S1, Table 1), mean yield under drought was compared among different recombinant classes: classes A, B, and C that possessed the N22 allele at nksdty1_1_30, and class D that lacked nksdty1_1_30 but possessed the N22 allele at adjacent marker nksdty1_1_34 and further down toward the telomere. Based on this analysis, there were two broad groups: group (1) with the N22 allele below the *sd1* gene (classes A+B+C+D) and group (2) with the Swarna allele below the *sd1* gene (classes E+F+G). The difference in mean grain yield (GY) under RS between these two groups was >1000 kg ha^−1^, confirming the presence of the *qDTY*_*1.1*_ gene below the *sd1* gene. Results of fine mapping suggested the QTL location to be downstream of the *sd1* gene, that is, between nksdty_1_34 and nksdty_1_38 (Fig. 3).

### Selection of dwarf drought-tolerant lines from the BIL population

After analyzing the *qDTY*_*1.1*_/*sd1* linkage and fine mapping, *qDTY*_*1.1*_ homozygous lines were developed with the dwarf allele of the *sd1* gene from this BIL population. Plants with double crossover events on both sides of the *sd1* gene were selected. Three dwarf *qDTY*_*1.1*_ homozygous lines (IR91659:41-95-5-B, IR91659:41-95-6-B, and IR91659:54-36-9-B) with plant height similar to that of Swarna under irrigated conditions showed a significant yield advantage over Swarna in the severe field drought experiment (Table 3A).

### Analysis of the *sd1*/*qDTY*_*1.1*_ region in traditional, pre-GR, and GR varieties

**Figure 4:**
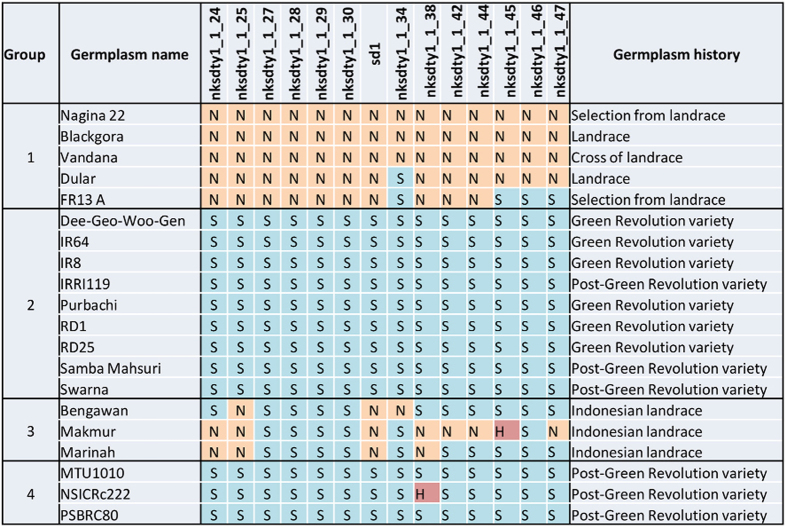
Graphical genotyping results presenting four groups of random genotypes with different combinations of the N22 (N) allele, Swarna (S) allele, or heterozygous (H) within the *qDTY*_*1.1*_ region; Group 1: pre- or GR drought-tolerant varieties having the tall *sd1* allele; Group 2: GR varieties having the dwarf allele of *sd1* with drought-sensitive (S) alleles in the *qDTY*_*1.1*_ region; Group 3: Indonesian landraces of East Java (rainfed environment) with the tall allele of the *sd1* gene but with low yield potential; Group 4: GR varieties that are semi-dwarf but preferred in rainfed areas because of high yield potential.

Association analysis using a random variety set revealed a significant association of markers within *qDTY*_*1.1*_ with DTF, GY, HI, and PH. The *sd1* gene was found to be significantly associated with PH but not with GY under RS (Table 4). Four different varietal groups were identified among 123 random genotypes that were genotyped for *sd1* and *qDTY*_*1.1*_ markers and compared for phenotypic variation for GY (Tables S2-S4) and related traits among medium- and late-duration groups (Fig. 4). Group 1 represents drought-tolerant genotypes that are either pre-GR varieties or landrace derivatives such as N22 and Vandana. This group has the tall allele of *sd1* along with the partial/full region of *qDTY*_*1.1*_. Group 2 is composed of the GR varieties with the dwarf allele of *sd1* and a Swarna-type *qDTY*_*1.1*_ region. Group 3 comprises lines that have the tallness allele of the *sd1* gene but the drought-sensitive allele of *qDTY*_*1.1*_ (all three varieties have the Swarna allele at marker locus nks1_1_30). Group 4 represents GR varieties that had neither the *sd1* tall allele nor the N22 allele of *qDTY*_*1.1*_. These are semi-dwarf varieties that show higher yield in rainfed areas^5^. The *sd1* profile of genotypes in Groups 3 and 4 indicates that the *sd1* gene is not responsible for high grain yield under drought and the performance of genotypes in Group 4 under drought is also not due to *qDTY*_*1.1*_. Though not absolutely, comparative analysis of all four groups presented in Fig. 4 indicates the responsiveness of *qDTY*_*1.1*_ and the non-responsiveness of *sd1* to drought tolerance.

### Analysis of *qDTY*_*1.1*_ and *sd1* using 3K Rice Genome sequence data

**Figure 5:**
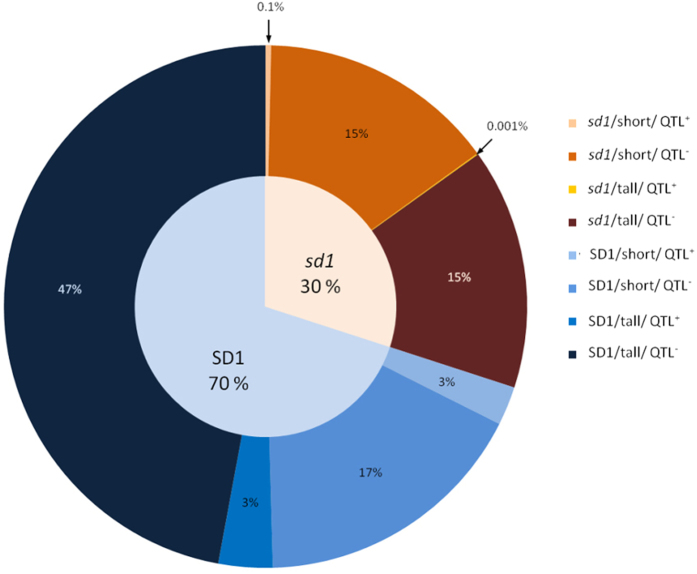
The relationship of culm length to the *sd1/SD1* haplotype with the presence of the N22 *qDTY*_*1.1*_ haplotype. From a total of 3000 genotypes, 1451 had data available for culm length. Numbers represent percentages in relation to *sd1/SD1* classification. Seventeen percent of the *SD1* tall genotypes were associated with the N22 haplotype and 99.9 percent of the *sd1* genotypes were not associated with the N22 haplotype, thus confirming the hypothesis that the selection for short plant type also selected for drought susceptibility alleles. Additional sources of dwarfism and drought tolerance are the most likely explanations for the discrepancies noted in this classification.

A linkage between the *sd1* allele and *qDTY*_*1.1*_ in the tall landraces, most of which are drought tolerant, is further supported through SNP analysis of the recently available 3K Rice Genome Project^19^. The relationship of culm length with type of cultivar revealed that GR advanced breeding lines and released varieties comprised 62% of the genotypes that have a culm length within 50 to 90 cm. In contrast, 90% of the genotypes with >90 cm culm length were landraces. Semi-dwarf plants in which one of the two well-known *sd1* alleles (LOC_Os01g66100; L266F)^20^ was present were represented by only 0.1% of the genotypes. However, there was 100% association between dwarf genotypes (<50 cm) and a novel mutation (Y342*) that leads to a termination codon in the third exon of LOC_Os01g66100 encoding gibberellin-20 oxidase. Also, a haplotype with three mutations in intron 2 was significantly related to semi-dwarfism. The one known and two novel alleles of *sd1*, fully associated with the semi-dwarf phenotype, were not linked to the putatively drought-tolerant N22 haplotype of 58 downstream genes spanning *qDTY*_*1.1*_. In contrast, in 17% of the tall genotypes (WT *gibberellin-20 oxidase;* LOC_Os01g66100), the haplotype of the 58 downstream genes was significantly similar to the N22 haplotype, the putative source of drought tolerance (Fig. 5). These results reinforced the hypothesis that the *sd1* gene was linked to drought-sensitive *qDTY*_*1.1*_ alleles.

### Physiological basis of the drought tolerance effect of *qDTY*_*1.1*_

**Figure 6:**
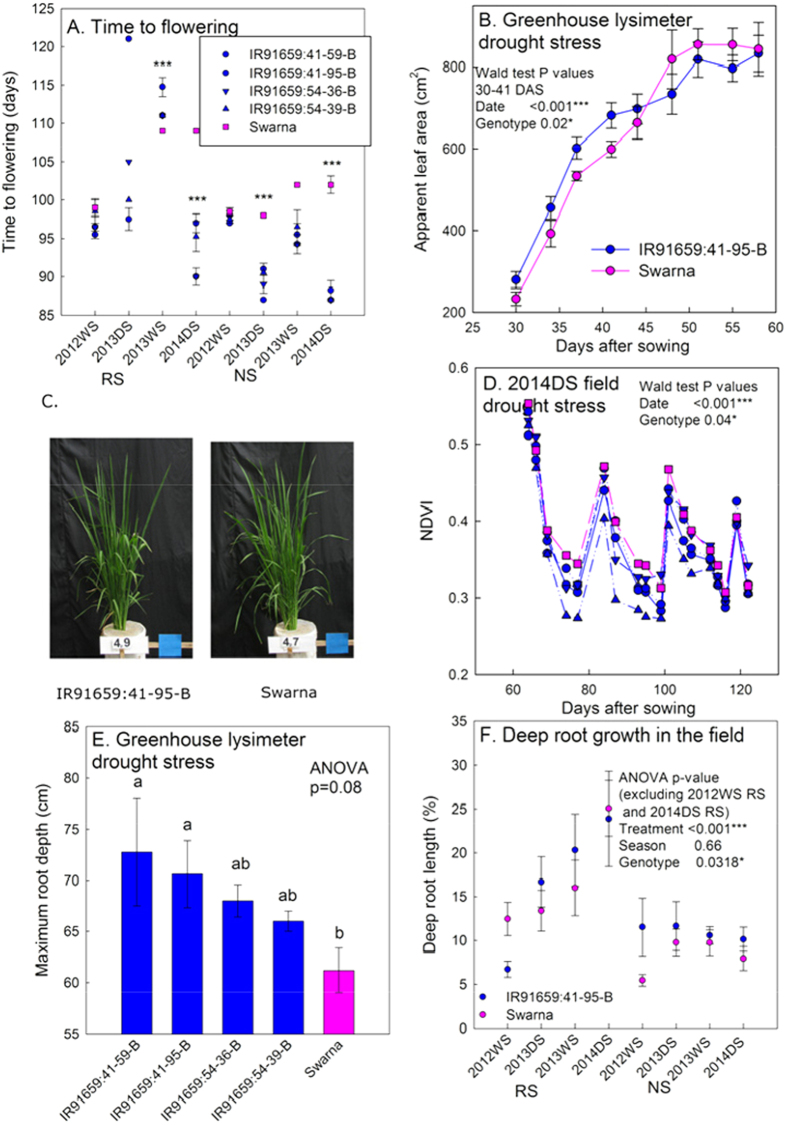
Physiological characterization of four selected *qDTY*_*1.1*_ BILs. (**A**) Flowering time across all field physiological studies, (**B**) apparent leaf area in the drought stress treatment of the greenhouse lysimeter experiment, (**C**) shoot images at 49 days after planting from the greenhouse lysimeter experiment, (**D**) normalized difference vegetation index (NDVI) in the 2014 DS field drought stress treatment (in which stress began at 60 days after sowing), (**E**) maximum root depth at harvest (54 days after planting) in the greenhouse lysimeter experiment, and (**F**) percent of root length at depth (below 30 cm) in the soil cores across all field studies. (RS; reproductive-stage drought stress, NS; non-stress control).

In detailed physiological studies on four drought-tolerant recombinant BILs in the Swarna background, the four recombinant BILs and Swarna showed a varying levels of flowering times under well-watered (NS) and drought stress (RS) conditions, with a significantly shorter flowering time observed in the *qDTY*_*1.1*_ BILs than in Swarna during the 2012 wet season (WS) and 2014 dry season (DS) under drought stress and in the 2013 DS and 2014 DS in the well-watered control (Fig. 6A). Drought stress was imposed early enough around 30 days after transplanting to minimize the effect of early DTF on drought stress. Swarna did not flower in the drought stress treatment in the 2013 DS. The aboveground shoot mass of *qDTY*_*1.1*_ BILs showed a dynamic response to the timing and severity of drought stress. In a greenhouse lysimeter study, *qDTY*_*1.1*_ BIL IR91659:41-95-B showed higher apparent leaf area than Swarna early in the drought stress treatment (Fig. 6B), but leaf area was similar among genotypes in the well-watered control (Figure S2-A). The height and plant type of the *qDTY*_*1.1*_ BILs were similar to those of Swarna (Fig. 6C). In the field, *qDTY*_*1.1*_ BILs showed a lower normalized difference vegetation index (NDVI) than Swarna when the stress treatment began early (2014 DS; Fig. 6D), and a higher NDVI than Swarna early in the drought stress treatment when the stress treatment began late (2013 DS; Figure S2-B). Starting from the reproductive stage, the *qDTY*_*1.1*_ BILs showed a greater allocation to stem mass as a proportion of the aboveground biomass in the field under drought and in the well-watered control (Figure S2-C, D) in the 2014 DS. The allocation to deep root growth was higher in the *qDTY*_*1.1*_ BILs than in Swarna. The *qDTY*_*1.1*_ BILs also showed higher maximum root depth in the drought stress treatment of the greenhouse lysimeter study (Fig. 6E) but not in the well-watered control (Figure S2-E). The BIL IR91659:41-95-B showed a higher proportion of root length at depth across seasons and treatments (Fig. 6F), except in the seasons when the drought stress was very severe (2012 WS and 2014 DS). The water uptake rates of *qDTY*_*1.1*_ BILs in the drought treatment of the greenhouse lysimeter study were closely related to leaf area patterns across the study (Figure S2-F).

### Linkage of other *DTY* QTLs with traits lost during the GR era

Similar to *qDTY*_*1.1*_, three other drought-yield QTLs were observed to show linkage with traits selected against during the GR. *qDTY*_*6.2*_ was linked with tall plant height, *qDTY*_*3.1*_ was linked with reduced grain yield under irrigated conditions, and *qDTY*_*3.2*_ was linked with very early days to flowering. The presence of the *qDTY*_*6.2*_ donor (IR55419-04) allele at three marker loci (RM3, RM541, and RM275) within *qDTY*_*6.2*_ led to increased yield under drought. Recombinant classes identified from a mapping population showed an association of RM275 with tall plant height within this region (Figure S3-A). A height difference of up to 4 cm under non-stress conditions and up to 6 cm under stress was observed for lines with the IR55419-04 and TDK1 alleles at this marker locus. For *qDTY*_*3.1*_, three marker loci (RM520, RM416, and RM16030) were associated with increased yield under drought in an Apo/Swarna population^4^. However, class analysis based on the three marker loci within this QTL showed that the presence of the drought-tolerant Apo allele at RM16030 led to a decline in yield under irrigated conditions (Figure S3-B). The presence of the drought-tolerant *qDTY*_*3.2*_ allele from Vandana at three marker loci (RM7332, RM523, and RM545) within *qDTY*_*3.2*_ was related to a reduction in DTF (Figure S3-C), which led to early maturity. Although this earliness was advantageous under severe drought stress, a flowering time of less than 80 days decreases yield potential under well-watered conditions. This has been the main reason that the highest-yielding GR varieties were bred to mature after more than 110 days; thus, this QTL was rejected during the development of GR varieties. Class analysis revealed the association of RM523 with early DTF.

In this study, recombinant lines with the drought tolerance alleles of *qDTY*_*6.2*_*, qDTY*_*3.1*_, and *qDTY*_*3.2*_ with desired height, yield, and DTF were identified. The dwarf lines with *qDTY*_*6.2*_ with plant height similar to that of TDK1 under irrigated conditions showed higher yield than TDK1 under severe field drought experiments (Table 3B). Lines with *qDTY*_*3.1*_ showed similar yield to Swarna under irrigated conditions and a significant yield advantage over Swarna under severe field drought (Table 3C). Similarly, lines with *qDTY*_*3.2*_ with later DTF than Vandana showed yield under drought similar to that of drought-tolerant cultivar Vandana, the donor of the *qDTY*_*3.2*_ QTL (Table 3D).

### Proportion of *qDTY* alleles in traditional and GR varieties

**Figure 7:**
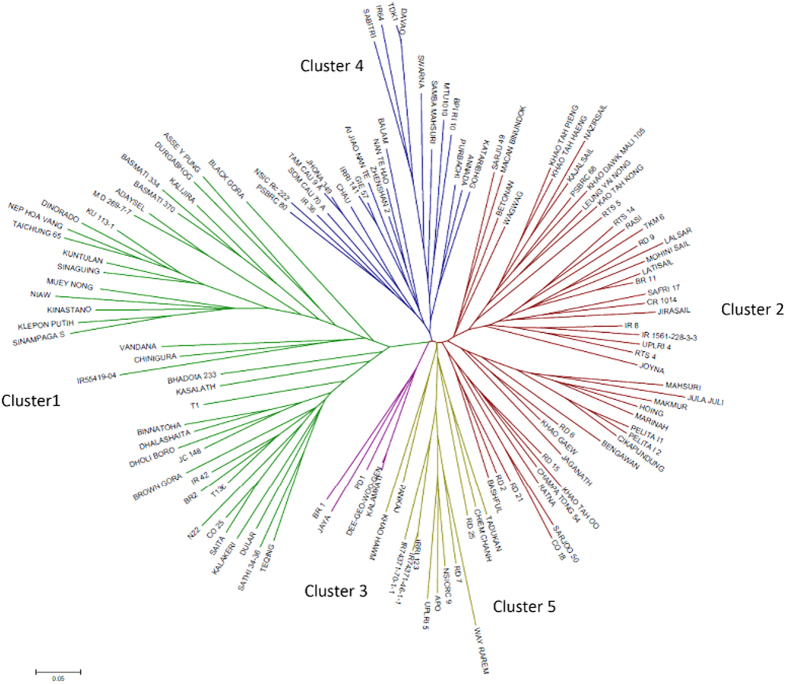
Neighbor-joining tree constructed based on C.S. Chord (Cavalli-Sforza and Edwards, 1967) for 11 *qDTY* regions. Clusters 1–5 show classification of 132 rice genotypes based on the allelic diversity in these regions.

**Figure 8:**
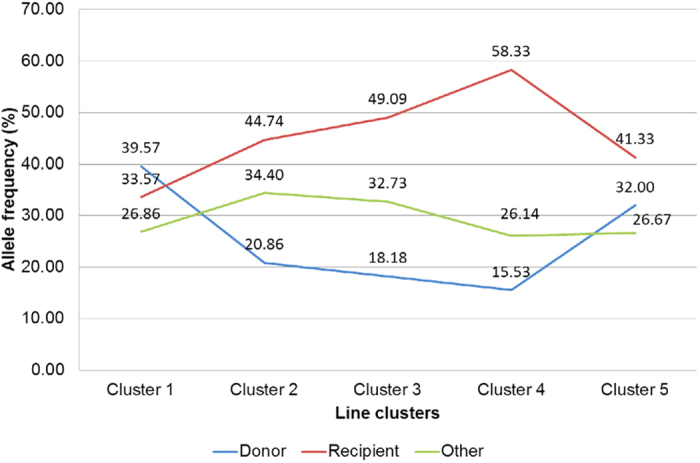
Allelic frequency patterns at 11 different *DTY* QTLs across traditional varieties, drought-tolerant donors, and modern high-yielding GR varieties. Clusters 1–5 correspond to the clusters identified in the diversity analysis.

Genetic diversity analysis for *qDTY* alleles conducted with 65 SSR markers in and around the 11 different *DTY* QTLs separated the drought-tolerant lines from the drought-susceptible lines into different clusters (Fig. 7). It also separated the traditional drought-tolerant donors (Cluster 1) with recently developed drought-tolerant varieties (Cluster 5) and further separated GR varieties into three distinct groups: early GR varieties represented by IR8 (Cluster 2), mid GR varieties represented by Jaya (Cluster 3), and late GR varieties represented by presently cultivated varieties IR36, IR64, Swarna, MTU1010, and Sambha Mahsuri (Cluster 4) (Fig. 7). Cluster 5 included the drought-tolerant varieties developed during the post-GR era: Apo, RD7, IR74371-46-1-1, and IR74371-70-1-1. The only exception in this group is Way Rarem, the cultivar which in itself is drought susceptible but has contributed the alleles for *qDTY*_*12.1*_. The proportion of *qDTY* tolerant, sensitive, and other unknown alleles at 65 SSR marker loci of 11 *qDTY* QTLs showed the highest percentage of donor alleles in traditional upland drought-tolerant varieties (Cluster 1, 40%), followed by recently developed varieties (Cluster 5, 32%). Among the GR varieties, the proportion of drought-tolerant alleles declined to 21% in Cluster 2, 18% in Cluster 3, and 16% in Cluster 4 varieties (Fig. 8). Interestingly, the frequency of the recipient allele (contributed by drought-susceptible GR varieties) increased from 34% in traditional donor varieties to 45% and 49% in early and mid GR varieties (Clusters 2 and 3), respectively, and to 58% in late GR varieties (Cluster 4). In recently developed drought-tolerant varieties, the frequency of the recipient allele decreased to 41% (Fig. 8).

## Discussion

The characterization of *qDTY*_*1.1*_ and *sd1* conducted in this study in terms of genetic linkage, presence in varieties with known drought tolerance, association with dwarfing genes, and physiological drought response, as well as the observed linkage of other *qDTY* with undesirable traits and the absence of *qDTY* alleles in GR varieties, strongly suggest that the GR did indeed select against drought tolerance in rice.

Multiple approaches confirmed that *qDTY*_*1.1*_ and *sd1* are linked, indicating their distinct effects on plant performance (*qDTY*_*1.1*_ on drought tolerance and *sd1* on plant height) and therefore the likelihood that semi-dwarf plant height could have been selected at the expense of drought tolerance in developing GR varieties. Testing the hypothesis on whether the *sd1* gene and *qDTY*_*1.1*_ are linked required four variables (plant height, GY under drought, presence of the *sd1* gene, and markers for *qDTY*_*1.1*_) to segregate in a genetically defined population. In the first test for linkage vs pleiotropy conducted through multiple trait–multiple interval mapping (MT-MIM) analysis on the RIL populations in which *qDTY*_*1.1*_ was originally identified, the pleiotropic effect obtained for GY–PH was found to be non-significant, suggesting a possible linkage between *qDTY*_*1.1*_ and *sd1* (Table 1). A similar approach has been followed previously by other researchers in testing the pleiotropic effects of co-located QTLs^21^. The re-mapping conducted in three N22-derived RIL populations in which *qDTY*_*1.1*_ was earlier identified^5^ suggested that *qDTY*_*1.1*_ is located downstream of the *sd1* gene (Fig. 1, Table 2). These results suggested the QTL location to be between *sd1* and RM431 (~500 kb). The position of *qDTY*_*1.1*_ was further validated to be distal to the *sd1* locus in a BC_3_F_4_ N22/Swarna-derived population fixed for the *sd1* dwarf allele, with similar PH and DTF, and that had high genetic background similarity. Recombination breakpoint analysis using a high-density custom SNP assay confirmed that the *qDTY*_*1.1*_ region was below the *sd1* gene, between nksdty1_1_34 and nksdty1_1_38 (Fig. 3). Association analysis conducted on a random set of varieties also revealed that the *sd1* gene has a significant association with PH but not with GY under RS (Table 4). A number of previous studies have suggested the possible linkage of *qDTY*_*1.1*_ and the *sd1* gene. Kumar *et al*. [16] identified a QTL in the *qDTY*_*1.1*_ region of a population that was not segregating for PH. Khowaja *et al*. [17] carried out a meta-QTL analysis that suggested linkage because the meta-QTL identified in that study was below the *sd1* gene, which is in agreement with the present study. Venuprasad *et al*. [18] also suggested the possible linkage of *qDTY*_*1.1*_ and the *sd1* gene in a study with five backcross-derived populations. In our study, re-mapping/fine mapping and association analysis as well as statistical analysis indicated that *qDTY*_*1.1*_ is located distal to the *sd1* gene, is tightly linked to it, and that the effect on increase in grain yield under drought contributed by this region is due to *qDTY*_*1.1*_ and not to *sd1*. The results from our study show a tight linkage between the tall allele of *sd1* and *qDTY*_*1.1*_. We subsequently broke this linkage and developed a population of dwarf BILs that was used for fine mapping of *qDTY*_*1.1*_. The observation that most popular high-yielding GR varieties have the drought-susceptible *qDTY*_*1.1*_ allele along with the dwarf *sd1* allele further suggests that the transfer of the dwarf allele of *sd1* led to the loss of the drought-tolerant *qDTY*_*1.1*_ allele in these varieties, resulting in their increased susceptibility to drought.

Breeding for the semi-dwarf trait that led to introgression of drought susceptibility was also revealed by the analysis of *qDTY*_*1.1*_ and *sd1* using the 3K Rice Genome sequence data. This susceptibility could be noticed only in recent years when such GR varieties showed a high reduction in yield due to increased occurrence of drought. Nevertheless, there are sufficient exceptions to the tall phenotype being linked to the N22 drought tolerance haplotype but very few exceptions to the short phenotype being linked to the drought susceptibility haplotype. Moreover, all genotypes analyzed for *sd1* linkage to the drought tolerance haplotype of N22 have not yet been phenotyped for response to drought. Therefore, the value of the data analyzed is restricted to addressing the hypothesis that the *sd1* gene is not causal to the tolerant haplotype of N22 genes comprising *qDTY*_*1.1*_. In that respect, our data support this hypothesis with 99.9% of the *sd1* dwarf genotypes not being linked to the drought-tolerant N22 *qDTY*_*1.1*_ haplotype and 17% of the *sd1* tall genotypes being linked to it (Fig. 5).

The physiological mechanisms related to the presence of *qDTY*_*1.1*_ further demonstrate that drought tolerance by this QTL is independent of plant height. It is likely that the mechanisms observed in the *qDTY*_*1.1*_ BILs – slightly earlier flowering time, a plastic shoot biomass response to drought, and the ability to increase root length at depth – act concertedly to confer higher yield under drought. The dynamic shoot mass response may be a growth regulation response to conserve water.

Of the candidate genes identified within the *qDTY*_*1.1*_ region (Table S5), NAM (no apical meristem protein) is one of the components of the drought-responsive NAC gene complex. The *SNAC1* gene in rice is a typical example of this family reported to increase spikelet fertility and seed setting rate under severe drought stress^22^. Another gene of this complex, *OsNAC10*, is reported to be responsible for root-specific expressions imparting drought tolerance and enhancing grain yield under drought^23^. One of the serine/threonine protein kinases in this fine-mapped region (LOC_Os01g66860) has been reported to show a differential response under drought stress in cultivar N22, which was used as the drought-tolerant parent in the present study^24^. A drought-responsive zinc finger protein factor has been isolated from rice cultivar N22^25^. Other examples of zinc finger proteins related to drought tolerance in rice are *ZFP252, Zat10/STZ*, and *WRKY* genes^26^,^27^,^28^. *STZ* was reported to increase spikelet fertility and grain yield under drought^27^. Further work in terms of differential expression analysis and/or transgenic validation of the candidate genes is necessary to pinpoint the gene(s) responsible for the increased drought tolerance conferred by *qDTY*_*1.1*_.

In addition to *qDTY*_*1.1*_, numerous other drought grain yield QTLs have been identified at the International Rice Research Institute (IRRI) during the past few years^4^,^5^,^12^,^13^,^29^,^30^,^31^. Some of these are reported to be associated with undesirable traits such as tallness (*qDTY*_*1.1*_ and *qDTY*_*6.2*_) and low yield under irrigated conditions (*qDTY*_*3.1*_). The *qDTY*_3.2_ QTL is associated with earliness, which often results in low yield potential. In all four of these cases, the characteristic traits of landraces such as tall plant height, low yield potential, and earliness are closely associated with the drought-tolerant *DTY* allele. In addition to studying the linkage between *sd1* and *qDTY*_*1.1*_, we investigated the association of *qDTY*_*6.2*_*, qDTY*_*3.1*_, and *qDTY*_*3.2*_ with the respective undesirable traits. The positive alleles of *qDTY*_*6.2*_, *qDTY*_*3.1*_, and *qDTY*_*3.2*_ were responsible for tall plant height, low yield under irrigated conditions, and very early maturity, respectively^4^,^5^,^30^,^32^. Indeed, most of the known drought-tolerant donors are intermediate/tall (more than 110–130 cm in lowland, 90–125 cm in upland) to tall (more than 130 cm in lowland, more than 125 cm in upland) in height, have low yield potential (less than 3.0 t ha^−1^), and mature early (80–100 days) compared with the GR varieties that are semi-dwarf (less than 110 cm in lowland, less than 90 cm in upland), have higher yield potential (more than 5.0 t ha^−1^), and show medium maturity duration (110–130 days). Therefore, the probable reason for traditional drought-tolerant varieties having characteristics such as tall plant height, lower yield potential, and early maturity is their linkages with QTLs for grain yield under drought (*qDTY*).

An allelic analysis with 11 *DTY* QTLs revealed a genetic shift in modern varieties with respect to these loci (Fig. 7). A sharp decrease in the frequency of drought-tolerant alleles in GR varieties as compared with the traditional drought-tolerant varieties/donors was observed (Fig. 8). Interestingly, the loss of drought-tolerant alleles continued as breeding for semi-dwarf varieties proceeded from IR8 in the 1960s to Jaya in the 1970s to IR36, IR64, Swarna, and Samba Mahsuri in the 1980s and beyond (Fig. 8). This trend appears to have resulted from the replacement of drought-tolerant alleles with alleles that were adapted to irrigated ecosystems through intercrossing among GR varieties, an approach that plant breeding has followed from 1966 until now. The linkage of drought-tolerant alleles with unfavorable traits/traits rejected for in the GR period (tall plant height, lower yield potential, and very early maturity) is likely the key factor for their loss during selection for semi-dwarf height, high yield potential, and medium maturity duration during the GR era.

In addition to the loss of alleles affecting grain yield under drought, another challenge to be addressed in improving the drought tolerance of modern rice varieties is the loss of alleles showing interactions with major alleles that either activate the drought tolerance response or increase the response of major drought tolerance alleles^30^,^33^. A significantly more detailed understanding of these interactions will be required before these aspects of drought tolerance can be improved.

The identification of undesirable linkages between alleles governing drought tolerance and tall plant height, lower yield under favorable conditions, or very early maturity was possible because of the strategic shift to work on QTLs for grain yield under drought from the previous strategy of identifying QTLs for secondary traits. Fortunately, the effect under drought is not a pleiotropic effect of the *sd1* gene or other such alleles as reported earlier^15^ but is the tight linkage between alleles imparting drought tolerance and alleles governing traits rejected for in developing GR varieties. This understanding provides a chance to eliminate these detrimental effects through careful breeding and preserving the drought tolerance imparted by these loci. The present study shows an example where the linkage between *sd1* and *qDTY*_*1.1*_ was systematically broken through the identification of double crossover recombinants to generate a large recombinant population and dwarf drought-tolerant lines possessing high yield potential were developed. Semi-dwarf drought-tolerant genotypes homozygous for *qDTY*_*1.1*_, dwarf plant height, and grain yield under favorable conditions similar to Swarna were successfully developed (Table 3). Recombinant lines without undesirable linkages related to *qDTY*_*3.1*_, *qDTY*_*3.2*_, and *qDTY*_*6.2*_ were also identified from the populations in which these were detected. In all four cases, plants with a significant yield advantage under drought over the recipient parents were developed without any compromise in yield potential under favorable conditions.

Conserved synteny predicts that, like rice, wheat and other cereals may also have lost important drought tolerance alleles because of similar undesirable linkages. The GR gene in wheat *Rht-b1* is located inside *qDSI*_*4B.1,*_ a major drought QTL^34^. Several QTLs were reported in the same region of wheat chromosome 4B^35^,^36^,^37^,^38^. The importance of the co-localization of drought tolerance QTLs with plant height loci was also revealed in sorghum^39^. Within *qDTY*_*1.1*_, a meta-QTL, *MqTL*_*1.1*_ was reported to show synteny with the chromosome 3 region near msu 2 in maize, with chromosome 4B near *Rht-b1* in wheat, and with the chromosome 6H region near *Bmac0316* in barley. The markers are reported to be linked to grain yield under drought in all three crops^40^. Loss of alleles adaptable to drought could also have occurred in other cereal crops wherein similar breeding procedures of selection for traits/alleles to increase yield under favorable conditions have been followed over decades. A comprehensive analysis involving all major cereal crops is required to investigate the allelic shift in modern breeding programs.

In conclusion, this work on fine mapping, physiological characterization, and linkage breaking of *qDTY*_*1.1*_ provides strong evidence that the tall *sd1* allele and *qDTY*_*1.1*_ loci are tightly linked and that the increased grain yield under drought conferred by *qDTY*_*1.1*_ is independent of *sd1* and plant height. The argument that GR variety development selected against drought tolerance in rice is further supported by the tight linkage between traits rejected for in the GR era and other *qDTY*, as well as by genetic diversity analyses. New drought-tolerant dwarf lines have been developed through the successful breakage of linkages between loci for drought tolerance and undesirable traits. This work provides new hope for developing high-yielding drought-tolerant dwarf varieties of rice and other cereal crops in order to extend the benefits of the GR to drought-prone regions.

## Materials and Methods

### Plant material and genotyping

#### Seven sets of plant materials were used

1. The previously identified *qDTY*_*1.1*_ region in three populations with 292 lines (N22/Swarna), 289 lines (N22/IR64), and 362 lines (N22/MTU1010) was genotyped with SNP markers underlying *qDTY*_*1.1*_ using the Fluidigm SNP genotyping platform (K-Biosciences Ltd.) for improved resolution of the map of the *qDTY*_*1.1*_*/sd1* region. RIL recombinants from the three populations were also genotyped with SNP markers flanking *qDTY*_*1.1*_ and with an *sd1* gene-specific marker.
2. An N22/4*Swarna backcross population was used for the *qDTY*_*1.1*_/*sd1* linkage and fine-mapping study. To develop this population, 217 semi-dwarf plants with height similar to that of Swarna were selected from 3000 BC_3_F_1_ plants. The selected plants were genotyped with *qDTY*_*1.1*_ markers RM11943, RM431, RM12023, RM12091, and RM12146. Two plants segregating for *qDTY*_*1.1*_ were identified and analyzed with the *sd1* gene-based marker to confirm the dwarf allele and were selfed. The resulting 180 BC_3_F_2:3_ N22/Swarna plants were analyzed with the *qDTY*_*1.1*_ markers. From the 180 BC_3_F_2:3_ N22/Swarna plants, 20 heterozygote recombinants for flanking markers for the *sd1* gene as well as *qDTY*_*1.1*_ were identified. These heterozygotes were selfed to produce ~1200 BC_3_F_4_ plants, which were then genotyped to select 160 recombinants based on the two flanking markers of *qDTY*_*1.1*_. In this BC_3_F_4_ N22/Swarna population, DTF, PH, and the *sd1* allele were similar in all selected lines. Background QTL effects were also neutralized in this population (Figure S1). Another QTL, *qDTY*_*3.2*_, identified in the N22/Swarna population^5^, was also fixed. Along with *qDTY*_*3.2*_, the five introgressed regions (RM246, RM278, RM335, RM434, and RM551) in the recombinant lines were fixed for the N22 allele and the population was segregating for only one (RM411) of the 120 markers used to test the background effect, which did not show significance for any of the tested drought-related traits. The fine-mapping study was conducted on these 160 recombinants (BC_3_F_4:5_ and BC_3_F_4:6_). Phenotypic variation for GY and related traits in the recombinant BIL population is presented in Table S1.
3. Four *qDTY*_*1.1*_ near-isogenic lines (NILs), IR91659-41-95-B, IR91659-41-36-B, IR91659-41-39-B, and IR91659-41-59-B, along with parents N22 and Swarna, were characterized in lysimeter studies as well as in field studies from 2012 to 2014 to understand the physiological mechanism of the drought tolerance conferred by *qDTY*_*1.1*_.
4. The effect of the *sd1* gene was analyzed in a set of 123 rice genotypes (Table S2), including landraces, traditional varieties, and cultivated varieties. These lines were genotyped with a panel of 29 SNP markers underlying *qDTY*_*1.1*_ with the Sequenom platform (described below). A gel-based analysis with the *sd1* gene-specific marker was carried out. SNP markers showing spurious calls were omitted and a total of 22 markers (21 SNPs + *sd1*) were used for analysis. This set of diverse genotypes was screened for GY under RS at IRRI in the 2012 DS and 2013 DS following the protocol described by Venuprasad *et al*. [41].
5. Culm length data were extracted from the SNP-Seek database^42^ for 3000 rice genotypes used to study the linkage pattern between the *sd1* allele and *qDTY*_*1.1*_ N22 allele (N22 being one of the 3000 genotypes sequenced). Varieties with missing culm length were eliminated from the analysis.
6. A BC_1_F_3_-derived IR55419-04/TDK1 (365 lines) population, a BC_1_F_4_-derived Apo/Swarna (490 lines) population, and an F_3_-derived Vandana/Way Rarem (242 lines) population were used to understand the linkage in the *qDTY*_*6.2*_*, qDTY*_*3.1*_, and *qDTY*_*3.2*_ regions, respectively. Genotypic data available for these populations^4^,^5^,^33^ were used to understand the linkages and to identify recombinants free from linkage drag within these populations (Figure S3).
7. In order to understand the allelic diversity present for 11 *qDTY* loci, a set of 132 diverse genotypes, including short and tall plant types of traditional drought-tolerant varieties, traditional drought-susceptible varieties, and modern rice varieties cultivated in rainfed and irrigated rice ecosystems, was used.

### Phenotyping under drought stress and irrigated conditions

The N22/Swarna BIL population was screened under lowland reproductive-stage drought stress (RS) and irrigated non-stress (NS) conditions in the 2012 DS and 2013 DS, whereas *qDTY*_*1.1*_ homozygote plants (N22/Swarna, N22/IR64, and N22/MTU1010) were screened in the 2012 DS at IRRI. The Apo/Swarna BC_1_-derived population was screened in the 2006 DS and 2007 DS under RS and NS conditions, respectively; Vandana/Way Rarem lines were screened under upland RS and NS conditions in the 2005 DS and 2006 DS; and the IR55419-04/TDK1 population was screened in the 2011 DS and 2012 DS under lowland RS and NS conditions, respectively. The RS and NS experiments were laid out in an alpha lattice design with two replications and managed as per the protocol described by Venuprasad *et al*.^41^ and Kumar *et al*.^43^.

For the physiological study, four BILs and parents N22 and Swarna were grown in a greenhouse lysimeter experiment from May to July 2013 in large lysimeters (95 cm tall × 20 cm diameter), as described by^44^. Five replicates of the two treatments were included in a completely randomized design. The soil in the lysimeters was maintained flooded until 20 days after planting, after which the lysimeters in the RS treatment were drained. The four *qDTY*_*1.1*_ BILs and parents were also characterized in the field during the 2012 WS (June 2012–October 2012), 2013 DS (December 2012–April 2013), 2013 WS (June 2013–October 2013), and 2014 DS (December 2013–April 2014) under transplanted lowland conditions, including NS and RS treatments with four replicates per treatment. Experimental plots were maintained flooded until 62, 75, 58, and 60 days after sowing (DAS) in the 2013 WS, 2013 DS, 2013 WS, and 2014 DS, respectively, after which irrigation in the RS treatment was stopped and rain was excluded using an automatic rainout shelter. The RS experiments were re-watered periodically as needed to sustain the plants until harvest. Soil water potential was monitored with one tensiometer per replicate installed at a depth of 30 cm in each RS experiment. The drought stress was most severe in the 2012 WS and 2014 DS, as indicated by the more negative soil water potential values for longer periods (Figure S4). Observations of the number of days to 50% flowering, plant height, biomass (BIO), grain yield, and harvest index were recorded as per the protocol described by Venuprasad *et al*.^4^.

All lysimeters were weighed three times per week to determine water uptake rates, at which time images of the shoot were acquired to measure apparent leaf area in Image J until plants were harvested at 60 days after sowing. Maximum root depth was determined as the distance from the base of the plant to the deepest root in the lysimeter. In the field, shoot growth was monitored by NDVI measured around mid-day using a GreenSeeker Handheld Sensor (NTech Industries, CA, USA) that was carried through the field (during the 2013 DS) or mounted about 1 m above the soil from a rack that rolled along the tracks of the rainout shelter (during the 2014 DS). Aboveground biomass partitioning was monitored throughout the 2014 DS, in which two hills per plot were harvested at each sampling. The leaf blades were separated from the sheaths and culms (the stems), and dry weight was determined. Root samples were taken at 128, 119, 113, and 101 DAS following re-watering of the RS treatment in the 2013 WS, 2013 DS, 2013 WS, and 2014 DS, respectively. Root samples were acquired using a 4-cm-diameter core sampler (fabricated at IRRI) to a depth of 60 cm (divided into 15-cm segments) and root length determined according Henry *et al*.^45^. ‘Percent deep roots’ was calculated as the root length below the soil depth of 30 cm as a percent of the total root length in the soil core.

### Genotyping with SSR markers of *DTY* QTLs

SSR genotyping of the N22-derived RILs and BILs was carried out for *qDTY*_*1.1*_. DNA of the populations was extracted from freeze-dried leaf samples that were cut in Eppendorf tubes and ground with a GENO grinder. Extraction was carried out by the modified CTAB method^46^. DNA samples were stored in 2-mL deep-well plates (Axygen Scientific, California, USA). DNA samples were quantified on 0.8% agarose gel and concentration adjusted to approximately 25 ng μL^−1^. For SSR analysis, PCR amplification was carried out with a 15-μL reaction mixture having 50 ng DNA, 1 × PCR buffer, 100 μM dNTPs, 250 μM primers, and 1 unit *Taq* polymerase enzyme. To resolve the PCR products, 8% non-denaturing polyacrylamide gels (PAGE) were used^47^. The set of 123 random genotypes was genotyped with 65 SSR markers across 11 *DTY* loci.

### Genotyping for the alleles of the *sd1* gene

Apart from phenotypic measurements for the presence of the *sd1* gene, the dwarfing allele of *sd1*, characterized by a 383-bp deletion spanning the first intron and second exon of the gene, was assayed by PCR amplification using primers flanking this deletion. The primers used were *sd1*-Forward: 5′-CACGCACGG GTTCTTCCAGGTG-3′ and *sd1*-Reverse: 5′-AGG AGAATA GGA GAT GGT TTA CC- 3′^48^. Product sizes for the dwarfing allele and the tallness allele were 348 and 731 bp, respectively. This region of the *sd1* gene is difficult to amplify consistently because of its high GC content; thus the PCR reaction was optimized for consistent amplification with minor modifications to the protocol of Masouleh^49^. For a 20-μl PCR reaction, 2 μl of 10x Enhancer buffer from Invtrogen (50 mM ), 1.2 μl of MgCl_2_ (50 mkM ), 3.0 μl of primers (5 pM each), 1 μl of dNTPs (2.5 mM each), 6 μl of 2X Enhancer, 0.2 μl of Platinum® *Taq* polymerase (5 U/μl), and 2.0 μl (20 ng/μl) of template were used. The thermocycling program consisted of initial denaturation at 94 °C for 5 min, followed by 45 cycles of amplification at 94 °C for 30 s, 55 °C for 30 s, and 72 °C for 1 min and a final extension at 72 °C for 3 min. The PCR product was visualized by electrophoresis in 2% agarose gel.

### Sequenom MassARRAY assay for fine mapping of *qDTY*_*1.1*_

A 29-plex Sequenom SNP assay was designed and validated for fine mapping of *qDTY*_*1.1*_ located between the 36.04- and 40.70-Mb region on the long arm of rice chromosome 1 (Fig. 2, Table S6). A total of 46 SNPs located in the 36.36- to 40.06-Mb region on chromosome 1 and polymorphic between parents N22 and Swarna (cf. OryzaSNP database; http://oryzasnp.plantbiology.msu.edu/) were taken for assay design using the Sequenom MALDI-TOF MassARRAY system^50^. The multiplex assay was designed using MassARRAY assay design 4.0 software and the reaction for a single tube multiplex containing 29 assays was optimized. The assay also included the *sd1* deletion locus with three possible SNP alleles, in addition to 28 SNPs located in 28 different genes in the QTL region (Fig. 2). After standardization of the primer concentrations for MALDI-TOF and validation of the assays in test samples, DNA samples of BILs showing recombination between *qDTY*_*1.1*_ and QTL flanking markers in a large N22/Swarna fine-mapping population and 46 random varieties were analyzed. All SNP calls worked well, except for one designed for the *sd1* del, for which only one of the alleles (T), representing the wild-type (tall) allele was called because of the high GC content of the amplicon resulting in poor amplification.

The primers were procured from IDT (Heverlee, Belgium). The iPLEX GOLD SNP genotyping was performed as per the manufacturer’s protocols and the genotype calls were analyzed using Sequenom Typer 4.0 software. The SNP calls in ACGT format generated for all the samples were converted to A, B, and H format for easy visualization of the recombinant break points. In cases of heterozygote calls (due to incomplete fixing of the inbred lines), a manual inspection of peak intensity was carried out to cross-check the validity of the calls. Data were analyzed using graphical genotyping of the *qDTY*_*1.1*_ region of the SNP calls from recombinant lines to identify the recombination break points and to find association with the phenotyping data.

### Statistical analysis

Statistical analysis was conducted using CROPSTAT v.4.2.3 (available at www.irri.org). Phenotypic means of entries were estimated using the following linear mixed model for the analysis of variance:


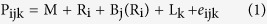


where P_ijk_ is the measurement recorded on a plot, M is the mean over all plots, and R, B, L, and e are replications, blocks, lines, and error, respectively. For estimating the entry means, replications and blocks within replicates were considered random whereas entries were fixed. While estimating the entry means across years, season effects were also taken as random.

Statistical analyses for the physiology experiments were performed in R v. 2.15.2^51^ by analysis of variance (ANOVA) (*aov* script) test for genotype and replication, and least significant difference (LSD) mean comparison was used as the post-hoc test. For measurements conducted on multiple dates, a repeated measures analysis was conducted with the mixed model ASREML using Wald’s test in R, with ‘genotype’ and ‘days after sowing’ as fixed variables and ‘replicate’ as a random variable.

### Multiple trait analysis

Multiple trait−multiple interval mapping (MT-MIM) analysis in the N22/IR64 and N22/Swarna RIL population was carried out as described by^21^. The analysis was performed with QGene software version 4.3.10 with 1000 permutations at significance level P < 0.01^52^. Combined mean phenotypic data of two years were used for the analysis. The model used for MT-MIM was


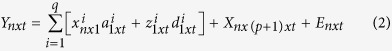


where q is the number of QTLs being fitted simultaneously, t is the number of analyzed traits, p is the number of non-genetic fixed factors, a is the additive effect, d is the dominant effect, X is the non-genetic fixed effect, B is the incidence matrix that links observation of the data with fixed effects, E is random error, and i = 1 − d.

### QTL analysis using additional markers in the *qDTY*_*1.1*_ region

Genetic map distances between markers were estimated according to their position on the physical map in reference to rice variety Nipponbare. One mega base pair (Mb) was assumed to be equivalent to ~3.92 cM^53^. The QTL analysis in different populations was performed using Inclusive Composite Interval Mapping (ICIM) software^54^. Results were also confirmed with QTL Network software v2.1 following a mixed model-based composite interval mapping method described by Yang *et al*.^55^. Candidate intervals were first selected, the significance of the association of intervals with the trait of interest was analyzed, and then the additive effect explained by the significantly associated interval was estimated. The significance level for determination of the candidate intervals, detection of putative QTLs, and their additive effects was P < 0.01.The F-value threshold for significance of the QTL was determined using 1000 permutation tests. The window size and walk speed used for the genome scan were 10 cM and 1 cM, respectively.

### Mapping of *qDTY*_*1.1*_ for improving resolution

A three-way approach was followed for the fine mapping of *qDTY*_*1.1*_ through N22-derived RILs, RIL recombinants, and N22/4*Swarna BIL populations. (i) In order to identify the break-point events between *sd1* and *qDTY*_*1.1*_, three SNP markers were added in and around *sd1*. One SNP was between RM11943 and *sd1* (id1024167) and two SNPs were between *sd1* and RM431 (id1024366 and id1024499). These markers were based on a panel of 44,000 SNPs published by Zhao *et al*.^56^. Genotyping was carried out using the Fluidigm SNP genotyping platform by K-Biosciences Ltd. QTL analysis was conducted on the phenotypic data from two field seasons. (ii) These additional SNPs were used to test the recombination between *sd1* and *qDTY*_*1.1*_. Recombinant lines among RILs between markers RM11943 and RM431 flanking both the *sd1* gene and *qDTY*_*1.1*_ locus were identified. The tight linkage between *sd1* and *qDTY*_*1.1*_ reduced the probability of double recombination in this interval; therefore, only the recombinant lines between the flanking markers were analyzed to find lines with break points between *sd1* and *qDTY*_*1.1*_. Further, because of a low number of recombinant lines in the individual populations, a pooled analysis of the 161 recombinant lines (48 from N22/Swarna, 57 from N22/IR64, and 56 from N22/MTU1010) from the three populations was carried out. Segregation for the *sd1* gene was analyzed using a functional marker as described above. (iii) The dwarf N22/Swarna RIL population was genotyped with SSR markers of *qDTY*_*1.1*_ (RM11943, RM431, RM12023, RM12091, and RM12146), SNP markers of *qDTY*_*1.1*_, as well as with the *sd1* gene-based indel marker. This population was homozygous for the dwarf *sd1* allele.

### Association analysis in a random set of varieties

Genotyping of 123 random varieties was conducted with genome-wide SNP markers within the *qDTY*_*1.1*_ region and an *sd1* gene-based functional marker. A genome-wide SNP panel was used to determine the population structure. A Sequenom MassARRAY multiplex assay was designed with 72 SNPs, including two wells of 36 plexi PLEX gold chemistry. These 72 SNPs represented 72 conserved single-copy rice genes and six genes/chromosomes, two genes each for all the telomeric and centromeric regions^57^. All SNPs were used for the population structure analysis as determined by STRUCTURE v.2.3.3 Software^58^. A burn-in period of 100000 with 10000 MCMC was followed. The *qDTY*_*1.1*_-specific SNP assay (Fig. 2) was used for marker-trait associations. Association of *qDTY*_*1.1*_ SNP markers as well as the *sd1* gene with drought-related traits was analyzed using population structure file, kinship matrix, and genotypic and drought-related phenotypic data of the 123 rice genotypes (Table S7) through TASSEL^59^ using the mixed linear model (MLM).

### Genetic diversity analysis of a set of diverse varieties

The genetic diversity analysis on the set of diverse varieties was conducted using the software PowerMarker V3.25^60^. Allele frequency was obtained using the bootstrap method with bootstrap number 10,000 and confidence interval 0.950. The frequency-based distance was obtained by the C.S. Chord method^61^. The neighbor-joining tree was constructed according to frequency-based distances obtained from PowerMarker using the software MEGA 5.2^62^.

### QTL class analysis

Recombinant classes were identified within *qDTY*_*3.1*_*, qDTY*_*3.2,*_ and *qDTY*_*6.2*_ based on the SSR marker data available for the three QTLs. Class means calculated based on the mean data of different recombinant classes were used to estimate the class effect on the respective phenotypic traits.

## Additional Information

**How to cite this article**: Vikram, P. *et al.* Drought susceptibility of modern rice varieties: an effect of linkage of drought tolerance with undesirable traits. *Sci. Rep.* **5**, 14799; doi: 10.1038/srep14799 (2015).

## Supplementary Material

Supplementary Information

## Figures and Tables

**Table 1:** The LOD values for pleiotropy effect of grain yield QTL *qDTY*_*1.1*_ on yield component traits using QGene software (combined analysis over two years under reproductive-stage stress (RS) and non-stress (NS) conditions).

Population	Environment	DTF	PH	BIO	HI
N22/IR64	NS	0.7(1.65)	0.9(2.42)	8.4(2.93)*	0.5(1.86)
	RS	1.8(2.12)	2.1(2.26)	6.7(2.96)*	0.9(1.92)
N22/Swarna	NS	1.6(1.84)	2.4(2.47)	3.7(3.21)*	1.5(1.69)
	RS	1.4(2.01)	1.6(2.41)	3.7(2.73)*	1.8(2.39)

Values in parentheses are LOD threshold values for significance. DTF = days to 50% flowering, PH = plant height, BIO=biomass, HI = harvest index.

^*^Significant pleitropic effect.

**Table 2:** The effect of linked markers of *qDTY*_*1.1*_ for grain yield under reproductive-stage drought stress in the three N22-derived RIL populations.

Population	Marker interval	F-value	Additive effect (%)	R^2^	Season
N22/Swarna	id1024366–id1024499	41	31	19.1	2009DS
	RM12091–RM12146	10	9	6.0	2010DS
	id1024366–id1024499	42	25	35.4	Combined
N22/IR64	id1024499–RM431	89	30	8.8	2009DS
	RM12091–RM12146	10	9	5.1	2010DS
	id1024499–RM431	85	26	9.1	Combined
N22/MTU1010	id1024366–id1024499	41	19	11.7	2009DS
	id1024366–id1024499	17	11	8.1	2010DS
	id1024366–id1024499	49	18	14.3	Combined
RIL recombinants	id1024366–id1024499	36	29	18.8	2009DS
	id1024366–id1024499	15	22	9.1	2010DS
	id1024366–id1024499	41	27	21.2	Combined

2009DS: 2009 dry season; 2010DS: 2010 dry season; Combined: combined analysis over the 2009 and 2010 dry seasons.

**Table 3:** High-yielding drought-tolerant recombinant lines for four *DTY* QTLs: *qDTY*_*1.1*_, *qDTY*_*6.2*_*, qDTY*_*3.1*_, and *qDTY*_*3.2*_.

QTL	Genotype	Stress			Non-stress		
		DTF	PH	GY	DTF	PH	GY
*A: qDTY*_*1.1*_	IR91659:41-95-B	90	72	3079	94	84	4530
	IR91659:54-36-B	92	68	3299	96	82	5027
	IR91659:41-95-B	86	69	3123	96	81	4487
	Swarna	102	63	561	96	84	4312
	LSD^0.05^	3	6	771	3	5	1246
*B: qDTY*_*6.2*_	IR90266-B-491-1	83	92	1750	74	109	5750
	IR90266-B-155-1	86	96	1637	73	113	5740
	IR90266-B-53-1	84	102	1947	70	116	5672
	TDK1	99	73	173	74	111	5985
	LSD^0.05^	4	10	714	8	14	1578
*C: qDTY*_*3.1*_	IR81896-B-B-309	98	82	1928	92	138	5174
	IR81896-B-B-481	98	76	929	95	127	5592
	IR81896-B-B-305	102	85	808	94	130	6717
	Swarna	—	54	0	103	82	4121
	LSD^0.05^	7	24	664	4	4	873
*D: qDTY*_*3.2*_	IR79971-B-102-B	79	84	920	83	110	3547
	IR79971-B-421-B	84	79	756	84	110	3156
	IR79971-B-86-B	68	76	847	73	101	3311
	Vandana	64	73	617	63	94	2180
	LSD^0.05^	5	10	241	3	12	782

LSD^0.05^: least significant difference at the 5% confidence level. DTF = days to 50% flowering, PH = plant height (cm), GY = grain yield (kg ha^−1^).

**Table 4:** Results of the association analysis in random rice genotypes for grain yield (GY) and yield-related traits − harvest index (HI), plant height (PH), and days to 50% flowering (DTF) − under reproductive-stage stress (RS) and non-stress (NS) conditions and combined analysis over two years (combined) with level of significance at 5% and 1%, respectively, denoted as *, **.

Trait	Year	Environment	Locus	Site	Significance	R^2^
DTF	2012	NS	nksdty1_1_46	39880000	*	0.06271
GY	2012	NS	nksdty1_1_44	39700000	**	0.09045
HI	2012	NS	nksdty1_1_20	37900000	**	0.08222
PH	2012	NS	nksdty1_1_25	38180000	*	0.05196
DTF	2012	RS	nksdty1_1_46	39880000	*	0.05987
PH	2012	RS	sd1	38330000	*	0.06308
DTF	2013	NS	nksdty1_1_34	38840000	*	0.0458
HI	2013	NS	nksdty1_1_47	39930000	*	0.07348
PH	2013	NS	nksdty1_1_46	39880000	*	0.06903
GY	2013	RS	nksdty1_1_34	38840000	**	0.05674
DTF	Combined	NS	nksdty1_1_46	39880000	*	0.06938
DTF	Combined	NS	nksdty1_1_40	39250000	*	0.05867
HI	Combined	NS	nksdty1_1_25	38180000	*	0.06236
PH	Combined	NS	nksdty1_1_46	39880000	*	0.06003
GY	Combined	RS	nksdty1_1_38	39080000	*	0.05159
PH	Combined	RS	sd1	38330000	*	0.07501

R^2^ is the phenotypic variance explained by the marker.
