# Supplementary material for: Drought susceptibility of modern rice varieties: an effect of linkage of drought tolerance with undesirable traits
Source: Sci Rep. 2015 Oct 13;5:14799. doi: 10.1038/srep14799 (PMC4602206; doi:10.1038/srep14799)
Supplement: Supplementary Information [file srep14799-s1.pdf]

Drought susceptibility of modern rice varieties: an effect of linkage of drought tolerance with undesirable traits

Prashant Vikram[1†], Mallikarjuna Swamy[1], Shalabh Dixit[1], Renu Singh[2], Bikram P. Singh[2], Berta Miro[1], Ajay Kohli[1], Amelia Henry[1], N.K. Singh[2] and Arvind Kumar[1]*

[1]Plant Breeding, Genetics, and Biotechnology Division, International Rice Research Institute, Los Baños, Philippines

[2]National Research Center for Plant Biology, Indian Agricultural Research Institute, New Delhi, India 110012

*Corresponding author e-mail: a.kumar@irri.org

[†]Present Address: Genetic Resource Program, Centro Internacional de Mejoramiento de Maíz y Trigo (CIMMYT), Km. 45 Carretera México-Veracruz, Col. El Batán, Texcoco, Edo. de Mex. 56130, México

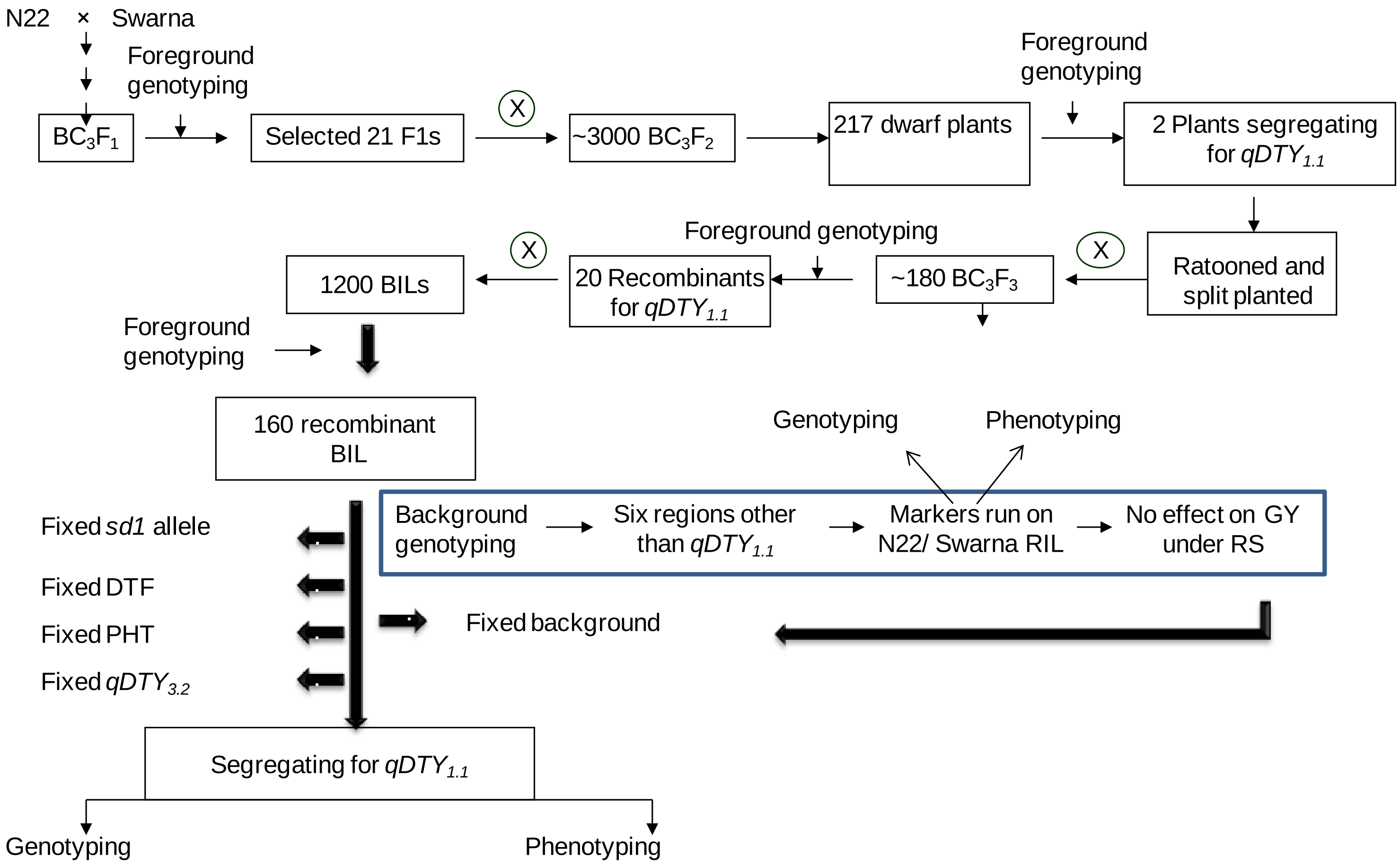

Figure S1: Development of the N22/Swarna BIL population with fixed *sd1* allele for semi-dwarf height and synchronized plant height (PH) and days to 50% flowering (DTF) similar to those of Swarna and minimized background effects.

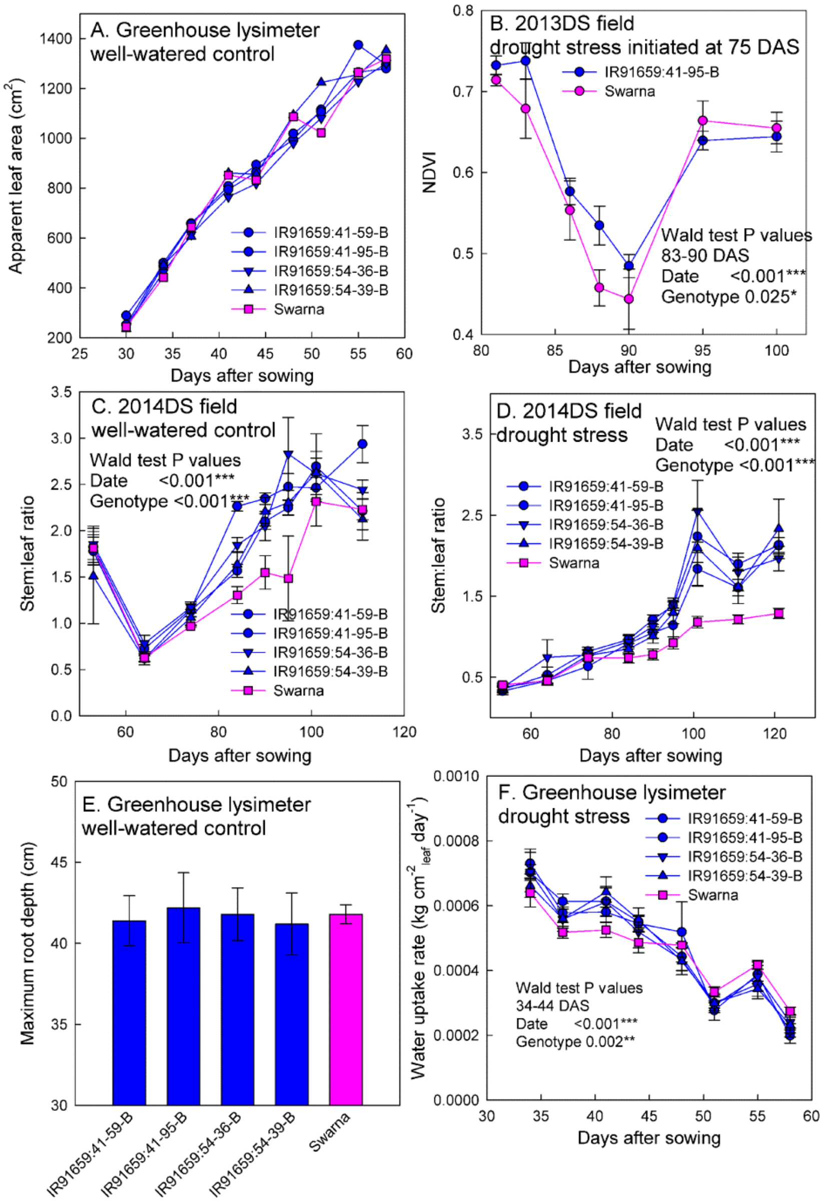

Figure S2: A) Apparent leaf area in the well-watered control treatment of the greenhouse lysimeter experiment, B) normalized difference vegetation index (NDVI) in the 2013 DS field drought stress treatment, C) stem:leaf ratio in the 2014 DS field well-watered control treatment, D) stem:leaf ratio in the 2014 DS field drought stress treatment, E) maximum root depth at the end of the study in the well-watered control treatment of the greenhouse lysimeter experiment, and F) water uptake rates in the drought stress treatment of the greenhouse lysimeter experiment.

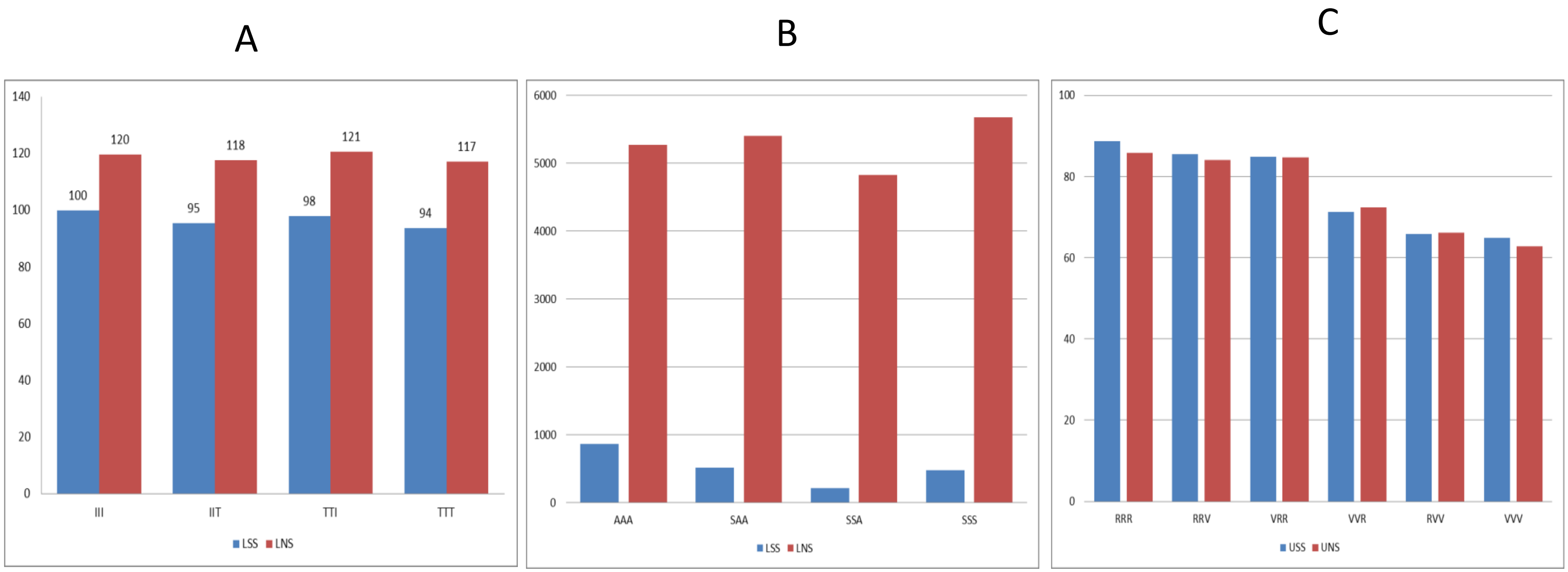

Figure S3: Class analysis with marker loci within (A) *qDTY*$_{6.2}$ (RM3, RM541, and RM275), (B) *qDTY*$_{3.1}$ (RM520, RM416, and RM16030), and (C) *qDTY*$_{3.2}$ (RM7332, RM523, and RM545), in IR55419-04/2* TDK1, Apo/3*Swarna, and Vandana/Way Rarem populations, respectively. Bar graphs show different degree of effects of recombinant classes on GY under non-stress conditions, DTF, and PH for the three QTLs, respectively.

qDTY$_{6.2}$ (RM3, RM541, and RM275): I, IR55419-04 allele; T, TDK1 allele;

qDTY$_{3.1}$ (RM520, RM416,l and RM16030): S, Swarna allele; A, Apo allele

qDTY$_{3.2}$ (RM7332, RM523, and RM545): V, Vandana allele; R, Way Rarem allele

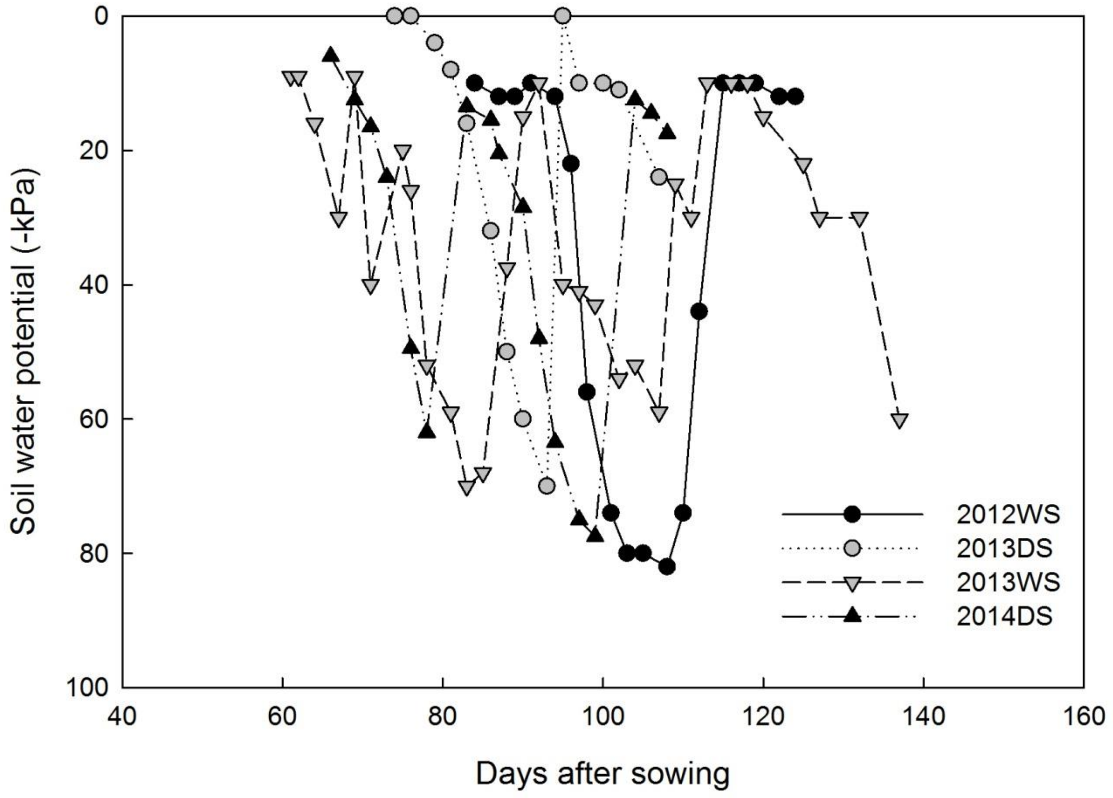

Figure S4: Soil water potential at a depth of 30 cm as measured by tensiometers in the drought stress treatments of the field physiology studies.

Table S1: Phenotypic variation for GY related traits under RS and NS in the dwarf N22/Swarna BIL population

	DTF (d)		PHT (cm)		BIO (kg ha^{-1})		GY (kg ha^{-1})		HI	
DS2012	NON STRESS	STRESS	NON STRESS	STRESS	NON STRESS	STRESS	NON STRESS	STRESS	NON STRESS	STRESS
MEAN ± SD	96 ± 7	100 ± 6	94 ± 6	72 ± 5	17790 ± 5988	10760 ± 7801	5448 ± 2160	1872 ± 696	0.296 ± 0.078	0.21 ± 0.13
RANGE	59 – 127	70 – 119	64 – 122	52 – 88	1628 – 33740	698 – 21040	557 – 11590	0 – 6869	0.043 – 0.50	0.001 – 0.44
LSD	12.7	6.3	9.9	9.9	12104	5615	4001	1304	0.2	0.3
DS2013										
MEAN ± SD	88 ± 6	92 ± 6	84 ± 6	67 ± 6	15490 ± 4539	9347 ± 2459	5341 ± 1530	1735 ± 624	0.401 ± 0.06	0.21 ± 0.09
RANGE	62 – 99	62 – 105	71 – 112	52 – 99	2450 – 33350	845 – 21650	368 – 10970	71 – 4238	0.105 – 0.499	0.001 – 0.50
LSD	5.4	5.6	7.3	9.7	180	9.7	516	315	0.82	0.25

Table S2: List of 123 genotypes/varieties used for the $qDTY_{1.1/}$ *sd1* association with GY under RS.

S.N.	Genotype/variety	S.N.	Genotype/variety	S.N.	Genotype/variety	S.N.	Genotype/variety	S.N.	Genotype/variety
1	AI JIAO NAN TE	26	CR 1014	51	JIRASAIL	76	MTU1010	101	RTS 12
2	ANNADA	27	DAVAO	52	JOYNA	77	MUEY NONG (WANG DIN)	102	RTS 14
3	APO	28	DEE-GEO-WOO-GEN	53	KAJALSAIL	78	N22	103	RTS 5
4	BALAM	29	DHAGAD DESHI	54	KALAKERI	79	NAN TE HAO	104	SAFRI 17
5	BASHFUL	30	DHALASAITA	55	KALAMKATI	80	NAZIRSAIL	105	SAMBA MAHSURI
6	BASMATI 334	31	DHOLI BORO	56	KALIJIRA	81	NIAW	106	SARJOO 50
7	BASMATI 370	32	DINORADO	57	KAO TAH KONG	82	NSIC RC 192	107	SARJU 49
8	BENGAWAN	33	DURGABHOG	58	KASALATH	83	NSIC RC 222	108	SATHI 34-36
9	BETONAN	34	GIE 57	59	KATARIBHOG	84	NSICRC 9	109	SMAGUING
10	BHADOIA 233	35	GS 529;DULAR	60	KHAO DAWK MALI 105	85	PANKAJ (IR 5-114-3-1)	110	SINAMPAGA SELECTION
11	BINNATOHA	36	HOING	61	KHAO GAEW	86	PELITA I 1	111	SOM CAU 70 A
12	BLACK GORA	37	IR 1561-228-3-3	62	KHAO HAWM	87	PELITA I 2	112	SWARNA
13	BPI RI 10	38	IR 36	63	KHAO TAH OO	88	PSBRC 68	113	T 1
14	BR 1	39	IR 42	64	KHAO TAH HAENG	89	PSBRC 80	114	T 136
15	BR 11	40	IR 64	65	KHAO TAH PIENG	90	PSBRC 82	115	TADUKAN
16	BR 2	41	IR 8	66	KU 113-1	91	PURBACHI (CHINESE 1)	116	TAICHUNG 65
17	BR 21	42	IR74371-46-1-1	67	LALSAR	92	RASI	117	TAICHUNG NATIVE 1
18	BROWN GORA	43	IR74371-70-1-1	68	LATISAIL	93	RATNA	118	TKM 6
19	CHAMPA TONG 54	44	IRRI 119	69	LEUANG YAI NONG	94	RD 1	119	UPLRI 4
20	CHAU	45	IRRI 123	70	MACAN BINUNDOK	95	RD 15	120	UPLRI 7
21	CHIEM CHANH	46	IRRI 141	71	MAGAWK DONG 269-7-7 (9)	96	RD 2	121	VANDANA
22	CHINIGURA	47	JAGANATH	72	MAHSURI	97	RD 25	122	WAGWAG
23	CIKAPUNDUNG	48	JAYA	73	MAKMUR	98	RD 6	123	ZHENSHAN 2
24	CO 18	49	JC 148	74	MARINAH	99	RD 7		
25	CO 25	50	JHONA 349	75	MOHINI SAIL	100	RD 9		

Table S3: Phenotypic variation for grain yield (GY)-related traits under reproductive-stage stress (RS) and non-stress (NS) in random varieties of medium duration.

	DTF (d)		PHT (cm)		BIO (kg ha^{-1})		GY (kg ha^{-1})		HI	
DS2012	NON STRESS	STRESS	NON STRESS	STRESS	NON STRESS	STRESS	NON STRESS	STRESS	NON STRESS	STRESS
MEAN ± SD	72 ± 9	71 ± 9	111 ± 18	99 ± 15	13280 ± 5430	9063 ± 4045	3880 ± 1794	2534 ± 1108	0.344 ± 0.07	0.313 ± 0.09
RANGE	58 – 105	51 – 91	68 – 156	67 – 134	2835 – 30850	2650 – 33400	702 – 9278	363 – 5296	0.12 – 0.50	0.026 – 0.50
LSD	6.7	5.8	23	12	9298	4160	2391	1512	0.11	0.13
DS2013										
MEAN ± SD	81 ± 10	78 ± 8	100 ± 18	85 ± 15	12430 ± 4611	7052 ± 6394	3078 ± 1372	1178 ± 707	0.36 ± 0.09	0.39 ± 0.04
RANGE	61 – 109	63 – 103	68 – 140	49 – 117	3650 – 43500	550 – 49000	372 – 10850	0.1 – 2807	0.04 – 0.49	0.00001 – 0.50
LSD	7.3	5.1	15	21	10393	14044	1974	906	0.21	0.28

Table S4: Phenotypic variation for grain yield (GY)-related traits under reproductive-stage stress (RS) and non-stress (NS) in random varieties of late duration.

	DTF (d)		PHT (cm)		BIO (kg ha^{-1})		GY (kg ha^{-1})		HI	
DS2012	NON STRESS	STRESS	NON STRESS	STRESS	NON STRESS	STRESS	NON STRESS	STRESS	NON STRESS	STRESS
MEAN ± SD	83 ± 12	88 ± 14	126 ± 19	113 ± 18	15180 ± 6905	12130 ± 5052	3206 ± 1863	2156 ± 1222	0.234 ± 0.11	0.21 ± 0.11
RANGE	62 – 112	61 – 123	72 – 164	72 – 157	5204 – 40550	2300 – 38000	439 – 8120	14 – 5763	0.02 – 0.50	0.004 – 0.49
LSD	23	11	39	20	15582	9827	4172	1573	0.26	0.15
DS2013										
MEAN ± SD	76 ± 13	86 ± 12	119 ± 20	103 ± 15	7707 ± 3198	3761 ± 1797	4372 ± 2006	1379 ± 1271	0.35 ± 0.09	0.18 ± 0.14
RANGE	60 – 108	62 – 109	76 – 169	65 – 146	1946 – 17460	2100 – 16000	1319 – 8957	0 – 4711	0.07 – 0.49	0.001 – 0.50
LSD	15	7	27	15	4335	2184	3008	815	0.22	0.25

Table S5: List of the candidate genes at the distal end of the *sd1* gene and tightly linked with RM431 (38.4–39.0 Mb).

S.No.	feature	start	end	Locus name	Putative function
1	gene	38401207	38401533	LOC_Os01g66120	No apical meristem protein, putative, expressed
2	gene	38409201	38409365	LOC_Os01g66130	armadillo/beta-catenin repeat family protein, putative, expressed
3	gene	38424266	38424621	LOC_Os01g66140	plus-3 domain containing protein, putative, expressed
4	gene	38432869	38433670	LOC_Os01g66150	expressed protein
5	gene	38435460	38437074	LOC_Os01g66160	pentatricopeptide, putative, expressed
6	gene	38439863	38440460	LOC_Os01g66170	SNARE associated Golgi protein, putative, expressed
7	gene	38443627	38443810	LOC_Os01g66180	cytochrome c, putative, expressed
8	gene	38450202	38450501	LOC_Os01g66190	expressed protein
9	gene	38460094	38460564	LOC_Os01g66200	expressed protein
10	gene	38465531	38465598	LOC_Os01g66210	retrotransposon protein, putative, Ty3-gypsy subclass
11	gene	38474878	38475218	LOC_Os01g66230	csAtPR5, putative, expressed
12	gene	38475617	38475831	LOC_Os01g66240	mitochondrion protein, putative, expressed
13	gene	38481235	38484007	LOC_Os01g66250	S-locus-like receptor protein kinase, putative, expressed
14	gene	38484468	38485604	LOC_Os01g66260	expressed protein
15	gene	38489525	38490169	LOC_Os01g66270	AP2 domain containing protein, expressed
16	gene	38493839	38494134	LOC_Os01g66280	transcriptional regulator, putative, expressed
17	gene	38500880	38501056	LOC_Os01g66290	OsMADS21 - MADS-box family gene with MIKCc type-box, expressed
18	gene	38509123	38509734	LOC_Os01g66300	KH domain containing protein, putative, expressed
19	gene	38516417	38516517	LOC_Os01g66310	expressed protein
20	gene	38519987	38520259	LOC_Os01g66320	expressed protein
21	gene	38525642	38526409	LOC_Os01g66330	ATP-dependent Clp protease ATP-binding subunit clpX, putative, expressed
22	gene	38527961	38528185	LOC_Os01g66340	expressed protein
23	gene	38534823	38535329	LOC_Os01g66350	DUF647 domain containing protein, putative, expressed
24	gene	38541818	38542109	LOC_Os01g66360	2-C-methyl-D-erythritol 4-phosphate cytidylyltransferase, putative, expressed

25	gene	38557970	38558381	LOC_Os01g66379	expressed protein
26	gene	38559137	38559341	LOC_Os01g66400	expressed protein
27	gene	38561851	38561924	LOC_Os01g66410	expressed protein
28	gene	38570487	38570861	LOC_Os01g66420	PHD finger protein, putative, expressed
29	gene	38574115	38574132	LOC_Os01g66440	expressed protein
30	gene	38582407	38582574	LOC_Os01g66450	retrotransposon protein, putative, unclassified, expressed
31	gene	38587950	38588740	LOC_Os01g66460	retrotransposon protein, putative, Ty3-gypsy subclass, expressed
32	gene	38589984	38591752	LOC_Os01g66470	retrotransposon protein, putative, Ty3-gypsy subclass, expressed
33	gene	38594450	38594743	LOC_Os01g66480	expressed protein
34	gene	38611435	38612105	LOC_Os01g66490	no apical meristem protein, putative, expressed
35	gene	38623771	38623836	LOC_Os01g66500	phosphoribosylformylglycinamidine synthase, putative, expressed
36	gene	38627746	38628127	LOC_Os01g66510	MLO domain containing protein, putative, expressed
37	gene	38635322	38635651	LOC_Os01g66520	serine/threonine-protein kinase RIO-like, putative, expressed
38	gene	38637331	38638234	LOC_Os01g66530	ARGOS, putative, expressed
39	gene	38645466	38645708	LOC_Os01g66544	expressed protein
40	gene	38650518	38650715	LOC_Os01g66560	signal recognition particle 72 kDa protein, putative, expressed
41	gene	38655236	38656447	LOC_Os01g66570	ZOS1-19 - C2H2 zinc finger protein, expressed
42	gene	38661257	38661310	LOC_Os01g66580	RNA polymerase III RPC4 domain containing protein, expressed
43	gene	38676217	38676530	LOC_Os01g66590	DUF260 domain containing protein, putative, expressed
44	gene	38687370	38688097	LOC_Os01g66600	rhodanese-like, putative, expressed
45	gene	38692318	38692621	LOC_Os01g66610	serine/threonine-protein kinase receptor precursor, putative, expressed
46	gene	38696091	38696135	LOC_Os01g66620	glucosyltransferase, putative, expressed
47	gene	38699042	38699146	LOC_Os01g66630	S-domain receptor-like protein kinase, putative, expressed
48	gene	38704701	38704872	LOC_Os01g66640	S-domain receptor-like protein kinase, putative, expressed
49	gene	38716611	38716756	LOC_Os01g66650	expressed protein
50	gene	38718491	38719023	LOC_Os01g66660	expressed protein
51	gene	38723347	38724165	LOC_Os01g66670	expressed protein
52	gene	38729814	38729926	LOC_Os01g66680	S-domain receptor-like protein kinase, putative, expressed
53	gene	38736544	38737856	LOC_Os01g66690	ZIP4/SPO22, putative, expressed
54	gene	38745734	38745918	LOC_Os01g66700	beta-hexosaminidase precursor, putative, expressed

55	gene	38749035	38749487	LOC_Os01g66710	polygalacturonase, putative, expressed
56	gene	38752611	38752935	LOC_Os01g66720	NADP-dependent oxidoreductase, putative, expressed
57	gene	38759291	38759513	LOC_Os01g66730	exosome complex exonuclease RRP40, putative, expressed
58	gene	38759757	38759950	LOC_Os01g66740	inactive receptor kinase At1g27190 precursor, putative, expressed
59	gene	38767579	38768280	LOC_Os01g66750	retrotransposon protein, putative, unclassified, expressed
60	gene	38770126	38770260	LOC_Os01g66760	inactive receptor kinase At2g26730 precursor, putative, expressed
61	gene	38778041	38778137	LOC_Os01g66780	retrotransposon protein, putative, unclassified
62	gene	38785135	38786060	LOC_Os01g66790	retrotransposon protein, putative, unclassified, expressed
63	gene	38788306	38788417	LOC_Os01g66800	expressed protein
64	gene	38790403	38790510	LOC_Os01g66810	expressed protein
65	gene	38795480	38795636	LOC_Os01g66820	inactive receptor kinase At1g27190 precursor, putative, expressed
66	gene	38803740	38803908	LOC_Os01g66830	pectinacetylesterase domain containing protein, expressed
67	gene	38810316	38810451	LOC_Os01g66840	pectinacetylesterase domain containing protein, expressed
68	gene	38815050	38815179	LOC_Os01g66850	pectinacetylesterase domain containing protein, expressed
69	gene	38838619	38840001	LOC_Os01g66870	transposon protein, putative, CACTA, En/Spm sub-class, expressed
70	gene	38843725	38843862	LOC_Os01g66890	BTBZ1 - Bric-a-Brac, Tramtrack, and Broad Complex BTB domain with TAZ zinc finger and Calmodulin-binding domains, expressed
71	gene	38854092	38854277	LOC_Os01g66900	expressed protein
72	gene	38859999	38860214	LOC_Os01g66910	expressed protein
73	gene	38864020	38864322	LOC_Os01g66920	Ser/Thr protein phosphatase family protein, putative, expressed
74	gene	38887107	38887723	LOC_Os01g66940	kinase, pfkB family, putative, expressed
75	gene	38888805	38889245	LOC_Os01g66950	expressed protein
76	gene	38891151	38891518	LOC_Os01g66960	selenoprotein precursor, putative, expressed
77	gene	38897220	38897714	LOC_Os01g66970	zinc finger, C3HC4 type domain containing protein, expressed
78	gene	38902608	38902752	LOC_Os01g66980	expressed protein
79	gene	38909394	38909598	LOC_Os01g66990	expressed protein
80	gene	38915255	38915492	LOC_Os01g67000	membrane-associated 30 kDa protein, chloroplast precursor, putative, expressed
81	gene	38916092	38916366	LOC_Os01g67010	expressed protein
82	gene	38924789	38925118	LOC_Os01g67030	auxin-responsive protein, putative, expressed
83	gene	38928602	38930270	LOC_Os01g67040	OsRhmbd5 - Putative Rhomboid homologue, expressed

84	gene	38934590	38934770	LOC_Os01g67054	calreticulin precursor protein, putative, expressed
85	gene	38938902	38939459	LOC_Os01g67070	expressed protein
86	gene	38945620	38945638	LOC_Os01g67080	expressed protein
87	gene	38946700	38946868	LOC_Os01g67090	IQ calmodulin-binding motif domain containing protein, expressed

Table S6: The details of $qDTY_{1.1}$ custom SNP markers used for genotyping.

SNP_ID	2nd-PCRP	1st-PCRP	AMP_LEN	UP_CONF	MP_CONF	Tm(NN)	PcGC	PWARN	UEP_DIR	UEP_MASS	UEP_SEQ	EXT1_CALL	EXT1_MASS	EXT1_SEQ	EXT2_CALL	EXT2_MASS	EXT2_SEQ
nksdty1_1_01	ACGTTGGATGAAGCTGATGGAAAGAGGGAG	ACGTTGGATGCTGAATTATGAAACCGGAAC	106	94.2	75.1	52	57.9	D	R	5779.8	CTCCTAAGGTGGATCCTGC	G	6027	CTCCTAAGGTGGATCCTGCC	A	6107	CTCCTAAGGTGGATCCTGCT
nksdty1_1_02	ACGTTGGATGGACATGTATAGTGGCCTGAC	ACGTTGGATGGAAGGATACCTGGTCTTTTG	92	98.3	75.1	48.3	31.8	d	R	7307.8	ttTCAACACTGAGATTGATTCTTT	T	7579	ttTCAACACTGAGATTGATTCTTTA	C	7595	ttTCAACACTGAGATTGATTCTTTG
nksdty1_1_04	ACGTTGGATGCCTGCTGTACTCTGCTTTAG	ACGTTGGATGGCCAACAAGCAGAATTTCAAG	120	93	75.1	45.6	33.3	D	F	7633	taagTGAACAATCTGACTCATAATC	C	7880	taagTGAACAATCTGACTCATAATCC	T	7960	taagTGAACAATCTGACTCATAATCT
nksdty1_1_05	ACGTTGGATGTTATGATGCCACAGCCTCTC	ACGTTGGATGCAAGATGAAGACAGCAATAGG	101	94.5	75.1	46.1	43.8	D	F	5376.5	ccTGCTCCATCTGCATTT	C	5624	ccTGCTCCATCTGCATTTC	G	5664	ccTGCTCCATCTGCATTTG

nksdty1_1_06	ACGTTGGATGCGCCGACTATAGAGGGTTC	ACGTTGGATGAATCGCTGCAACGGTAACGG	103	94.5	75.1	46.8	50	DH	F	6238.1	gggcTAGAGGGTTCGCTGAA	C	6485	gggcTAGAGGGTTCGCTGAAC	T	6565	GCGACAGCTCCTTCATCTCCTCGC
nksdty1_1_07	ACGTTGGATGGTGCCCAAGGCCATCGTGC	ACGTTGGATGTAGAGGCGGTACTTCTGGAG	106	81.6	75.1	52.2	52.9	D	F	5170.4	ATCGTGCGCCTCATGAA	C	5418	ATCGTGCGCCTCATGAAC	T	5498	ATCGTGCGCCTCATGAAT
nksdty1_1_1	ACGTTGGATGAGGAGTTCCATGATCGTCAG	ACGTTGGATGCACGCACGGGTTCTTCCAG	116	89.7	75.1	47.3	53.3	D	R	6062	GCGACAGCTCCTTCATCTCCTCGC	G	6309	atcccTGATCGTCAGCGACAC	T	6333	atcccTGATCGTCAGCGACAA
nksdty1_1_10	ACGTTGGATGTTGTGCAGCTATGGAGGAAG	ACGTTGGATGTGAAACACCAATCCGGCAAG	117	98.9	75.1	46.4	56.2	d	F	5017.3	GTGTGTGCGGAGATAG	C	5265	GTGTGTGCGGAGATAGC	T	5344	GTGTGTGCGGAGATAGT
nksdty1_1_11	ACGTTGGATGGGCATGCTACTAGTAAAGAG	ACGTTGGATGTAACACCCAGTTAACGTGGC	113	97.9	75.1	50.9	40.9	D	R	6976.6	gCCCAACATATCGATCGAATTCA	T	7248	gCCCAACATATCGATCGAATTCAA	C	7264	gCCCAACATATCGATCGAATTCAG

nksdty1_1_13	ACGTTGGATGGGTTTCCATCAAAGCGGAAC	ACGTTGGATGTGCCGCGTCAAGCAACTCTC	97	94.3	75.1	57.7	63.2	Dh	R	6529.3	aaGGGAGCTGCACAGTGGACA	G	6776	aaGGGAGCTGCACAGTGGACAC	A	6856	aaGGGAGCTGCACAGTGGACAT
nksdty1_1_15	ACGTTGGATGGGTAGTTTTTGCTGCTGCTG	ACGTTGGATGCATCCAAGACCATACCATGC	97	100	75.1	46.1	53.3	d	F	4630	TGCATTCTGTGGGTG	C	4877	TGCATTCTGTGGGTGC	T	4957	TGCATTCTGTGGGTGT
nksdty1_1_18	ACGTTGGATGGGCTGCGATGTAGTTTAAGG	ACGTTGGATGATCTTGTTGCCTGTCTCCAC	117	98.9	75.1	46.6	44.4	d	R	5698.7	gACATCTCTCCCTTACTGA	G	5946	gACATCTCTCCCTTACTGAC	T	5970	gACATCTCTCCCTTACTGAA
nksdty1_1_20	ACGTTGGATGTATTGACAAAGGCACCCCTG	ACGTTGGATGACACCTACTTGAATGCAGAG	118	97.3	75.1	52.5	52.6	D	F	7070.6	ggatCATGCTTTGCCAGGTTGAC	A	7342	ggatCATGCTTTGCCAGGTTGACA	G	7358	ggatCATGCTTTGCCAGGTTGACG
nksdty1_1_21	ACGTTGGATGACTCCTCACCGTGTACTTGC	ACGTTGGATGTGTTGCGCGGCAAGATCAAG	109	96.8	75.1	50.6	66.7	D	R	4519.9	CTTGCCGGCGTCATC	G	4767	CTTGCCGGCGTCATCC	T	4791	CTTGCCGGCGTCATCA

nksdty1_1_23	ACGTTGGATGTGGTCGTAGAAGAACTCCTG	ACGTTGGATGATGAGCCTGTGGTGGATGGT	112	95.5	75.1	58.7	81.2	d	R	6178	acggCGGCGACGCCGACGAG	G	6425	acggCGGCGACGCCGACGAGC	T	6449	acggCGGCGACGCCGACGAGA
nksdty1_1_24	ACGTTGGATGCAACGAGAAAAACGACGCAC	ACGTTGGATGATTGCAGGTCCGGTGTCAGT	100	96.1	75.1	59.2	59.1		R	7810.1	gcccGCTCTCTTTGCACAGCCGCTTC	T	8081	gcccGCTCTCTTTGCACAGCCGCTTCA	A	8137	gcccGCTCTCTTTGCACAGCCGCTTCT
nksdty1_1_25	ACGTTGGATGCAGCTTCAACAATAGCAGCC	ACGTTGGATGGGGAAAAAATAGTCGTGCTC	100	98.6	75.1	56.9	55	D	F	6391.2	cGCCGCAATGCTTGAACCTGA	C	6638	cGCCGCAATGCTTGAACCTGAC	T	6718	cGCCGCAATGCTTGAACCTGAT
nksdty1_1_27	ACGTTGGATGGCCAGGTTTTGATGTATGAG	ACGTTGGATGGGCAAAGAGTGAAACGGTTG	115	97.7	75.1	45.5	38.9	D	F	5575.6	ATATCTTGGTGAGGTTGT	C	5823	ATATCTTGGTGAGGTTGTC	T	5903	ATATCTTGGTGAGGTTGTT
nksdty1_1_28	ACGTTGGATGCGGTGCATCATAGTCATAGC	ACGTTGGATGCCTTTTGAAGTACCATGGTG	105	98.5	75.1	47.4	60	Ds	R	4553	TCCAGTAGTGCGACC	T	4824	TCCAGTAGTGCGACCA	C	4840	TCCAGTAGTGCGACCG

nksdty1_1_29	ACGTTGGATGCAACATCCTGGTAATTCCCC	ACGTTGGATGCAATTCAAAAGACATGCTGGG	117	93.4	75.1	50.2	36.4	d	F	6684.4	TACCATTTTGCCAGATATGCTA	C	6932	TACCATTTTGCCAGATATGCTAC	T	7012	TACCATTTTGCCAGATATGCTAT
nksdty1_1_30	ACGTTGGATGTCTTTCCCAACTACGTACGC	ACGTTGGATGTGTGCAGTTAGCTAGTGCAG	99	100	75.1	48.7	52.9	D	F	5210.4	GAGCTTGGTGCAAACTC	C	5458	GAGCTTGGTGCAAACTCC	T	5538	GAGCTTGGTGCAAACTCT
nksdty1_1_34	ACGTTGGATGACAGCAGCATGTGATCAGAC	ACGTTGGATGGAAAACAGGGCGGTGATCTC	106	98.4	75.1	49.4	33.3	D	R	7399.8	CCAGATTTGGAGAATAATAATGCT	T	7671	CCAGATTTGGAGAATAATAATGCTA	A	7727	CCAGATTTGGAGAATAATAATGCTT
nksdty1_1_38	ACGTTGGATGCATGTTCAAATTATACCTGGG	ACGTTGGATGAATCATGACCTGGTAAATGG	107	81	75.1	49.8	34.8	h	F	8242.4	cttcTACCTGGGAAAAAAGTTCAATTC	A	8514	cttcTACCTGGGAAAAAAGTTCAATTCA	G	8530	cttcTACCTGGGAAAAAAGTTCAATTCG
nksdty1_1_40	ACGTTGGATGCAGAGCTTGACCTCGAGGAA	ACGTTGGATGCTACGGGATCTTCCTCGTC	80	90.7	75.1	54.2	64.7	dS	R	6899.5	gggctAGGAAGACGGCGACGAG	G	7147	gggctAGGAAGACGGCGACGAGC	C	7187	gggctAGGAAGACGGCGACGAGG

nksdty1_1_42	ACGTTGGATGCTACCAGAAAGCCTTGCTTG	ACGTTGGATGGTGTAATTTGGGAAGGTAGG	99	98.6	75.1	54.2	45.5	d	R	8397.5	gggtaAGCTGGGCATGAAAATGAACTC	G	8645	gggtaAGCTGGGCATGAAAATGAACTCC	A	8725	gggtaAGCTGGGCATGAAAATGAACTCT
nksdty1_1_44	ACGTTGGATGCTCAGAAACAGTAGAGACAG	ACGTTGGATGGAACAAGAGCTTCAGGAATC	101	97.1	75.1	45.8	33.3	D	F	7640	tctcAACAGTAGAGACAGATTTTTC	A	7911	tctcAACAGTAGAGACAGATTTTTCA	G	7927	tctcAACAGTAGAGACAGATTTTTCG
nksdty1_1_45	ACGTTGGATGTGTGGATGATAGTACTAGGC	ACGTTGGATGCAGCATTTCTTGCTTCCCAG	107	98.4	75.1	47.3	38.1	D	R	7763.1	gagtATAGTACTAGGCATGAAACAG	G	8010	gagtATAGTACTAGGCATGAAACAGC	C	8050	gagtATAGTACTAGGCATGAAACAGG
nksdty1_1_46	ACGTTGGATGGAATTCCAATGTGGTAGGCG	ACGTTGGATGTTTGCAGCAGTCAATGTGGG	83	98.9	75.1	55.2	50	d	R	8404.5	gtgcaAATGTGGTAGGCGGAATGACTC	T	8676	gtgcaAATGTGGTAGGCGGAATGACTCA	C	8692	gtgcaAATGTGGTAGGCGGAATGACTCG
nksdty1_1_47	ACGTTGGATGTGGATACCCTCTTCAGCAAC	ACGTTGGATGGAAACTGAAGCATAGTTCTC	119	93	75.1	50.8	29.6	D	F	8330.5	AGCAACATCTGTAGTAAATAGTAAGTT	C	8578	AGCAACATCTGTAGTAAATAGTAAGTTC	A	8602	AGCAACATCTGTAGTAAATAGTAAGTTA

Table S7: Fst values of the genotypes grouping 123 genotypes into four different subpopulations.

Genotype	Fst1	Fst2	Fst3	Fst4
AIJIAONANTE	0.013	0.114	0.008	0.865
ANNADA	0.003	0.018	0.015	0.965
Apo	0.005	0.19	0.01	0.795
BALAM	0.01	0.169	0.709	0.112
BASHFUL	0.008	0.548	0.02	0.424
BASMATI334	0.917	0.024	0.019	0.04
BASMATI370	0.856	0.022	0.065	0.057
BENGAWAN	0.004	0.971	0.007	0.018
BETONAN	0.013	0.91	0.031	0.046
BHADOIA233	0.011	0.012	0.966	0.011
BINNATOHA	0.004	0.011	0.975	0.01
Blackgora	0.003	0.004	0.989	0.004
BPIRI10	0.013	0.324	0.008	0.656
BR1	0.005	0.007	0.982	0.006
BR11	0.005	0.59	0.016	0.389
BR2	0.005	0.06	0.891	0.044
BR21	0.006	0.278	0.008	0.707
Browngora	0.003	0.003	0.99	0.004
CHAMPATONG54	0.006	0.965	0.005	0.023
CHAU	0.02	0.056	0.006	0.918
CHIEMCHANH	0.011	0.017	0.009	0.963
CHINIGURA	0.008	0.92	0.015	0.057
CIKAPUNDUNG	0.004	0.974	0.005	0.017
CO18	0.006	0.008	0.977	0.009
CO25	0.005	0.008	0.978	0.008
CR1014	0.008	0.782	0.037	0.173
DAVAO	0.011	0.655	0.212	0.122

DEE-GEO-WOO-GEN	0.003	0.085	0.007	0.905
Dhagaddeshi	0.008	0.313	0.303	0.376
DHALASAITA	0.014	0.043	0.923	0.021
DHOLIBORO	0.006	0.005	0.984	0.005
DINORADO	0.988	0.004	0.004	0.003
DURGABHOG	0.979	0.008	0.006	0.007
GIE57	0.007	0.319	0.009	0.665
DULAR	0.004	0.01	0.977	0.009
HOING	0.004	0.942	0.005	0.049
IR156122833	0.004	0.631	0.009	0.356
IR36	0.011	0.011	0.967	0.011
IR42	0.004	0.062	0.791	0.143
IR64	0.007	0.064	0.012	0.917
IR8	0.004	0.104	0.007	0.885
IR743714611	0.037	0.235	0.013	0.714
IR743717011	0.005	0.081	0.007	0.908
IRRI119	0.003	0.056	0.006	0.935
IRRI123	0.005	0.352	0.008	0.634
IRRI141	0.004	0.318	0.005	0.673
JAGANATH	0.006	0.647	0.068	0.279
JAYA	0.004	0.519	0.041	0.436
JC148	0.003	0.009	0.979	0.009
JHONA349	0.012	0.452	0.164	0.372
JIRASAIL	0.009	0.314	0.156	0.522
JOYNA	0.011	0.676	0.011	0.302
KAJALSAIL	0.03	0.352	0.549	0.068
Kalakeri	0.003	0.004	0.987	0.005
KALAMKATI	0.004	0.076	0.018	0.902
KALIJIRA	0.972	0.009	0.009	0.01
KAOTAHKONG	0.008	0.929	0.009	0.054
KASALATH	0.004	0.048	0.625	0.322

KATARIBHOG	0.003	0.122	0.011	0.863
KHAODAWKMALI105	0.006	0.967	0.007	0.02
KHAOGAEW	0.006	0.87	0.024	0.099
KHAOHAWM	0.005	0.323	0.019	0.653
KHAOTAHOO	0.014	0.884	0.004	0.097
KHAOTAHHAENG	0.015	0.801	0.034	0.149
KHAOTAHPIENG	0.006	0.942	0.006	0.046
KU1131	0.006	0.915	0.008	0.072
LALSAR	0.015	0.455	0.111	0.419
LATISAIL	0.003	0.936	0.007	0.053
LEUANGYAINONG	0.008	0.966	0.005	0.021
MACANBINUNDOK	0.005	0.468	0.006	0.521
MAGAWKDONG26977(9)	0.987	0.004	0.004	0.005
MAHSURI	0.008	0.894	0.019	0.079
MAKMUR	0.003	0.971	0.004	0.022
MARINAH	0.003	0.934	0.013	0.05
MOHINISAIL	0.006	0.501	0.051	0.442
MTU1010	0.005	0.064	0.006	0.924
MUEYNONG(WANGDIN)	0.525	0.114	0.171	0.19
N22	0.003	0.004	0.987	0.005
NAN THE AO	0.004	0.177	0.014	0.805
NAZIRSAIL	0.004	0.966	0.005	0.025
NIAW	0.007	0.64	0.067	0.285
NSICRc192	0.007	0.263	0.009	0.72
NSICRc222	0.009	0.112	0.011	0.868
NSICRC9	0.004	0.242	0.013	0.741
PANKAJ(IR511431)	0.004	0.957	0.005	0.034
PELITAI1	0.004	0.969	0.005	0.022
PELITAI2	0.005	0.966	0.007	0.023
PSBRC68	0.003	0.047	0.006	0.944
PSBRC80	0.023	0.102	0.006	0.869

PSBRc82	0.036	0.674	0.046	0.243
PURBACHI(CHINESE1)	0.003	0.124	0.012	0.861
RASI	0.009	0.59	0.006	0.395
RATNA	0.003	0.045	0.005	0.947
RD1	0.003	0.093	0.006	0.897
RD15	0.005	0.669	0.019	0.306
RD2	0.003	0.774	0.012	0.21
RD25	0.003	0.54	0.006	0.451
RD6	0.006	0.686	0.017	0.291
RD7	0.005	0.852	0.01	0.133
RD9	0.023	0.131	0.137	0.709
RTS12	0.006	0.071	0.007	0.917
RTS14	0.013	0.829	0.037	0.121
RTS5	0.023	0.378	0.011	0.588
SAFRI17	0.019	0.495	0.013	0.473
Sambamahsuri	0.005	0.029	0.019	0.947
SARJOO50	0.007	0.04	0.011	0.942
SARJOO 49	0.005	0.7	0.007	0.288
SATHI-34-36	0.005	0.032	0.94	0.023
SMAGUING	0.983	0.004	0.005	0.008
SINAMPAGASELECTION	0.988	0.004	0.005	0.003
SOMCAU70A	0.006	0.241	0.016	0.737
SWARNA	0.006	0.553	0.013	0.428
T1	0.01	0.011	0.947	0.032
T136	0.008	0.06	0.893	0.039
TADUKAN	0.007	0.063	0.008	0.922
TAICHUNG65	0.005	0.007	0.981	0.007
TAICHUNGNATIVE1	0.004	0.007	0.982	0.007
TKM6	0.092	0.675	0.009	0.223
UPLRI4	0.006	0.204	0.009	0.781
UPLRi7	0.007	0.585	0.004	0.405

Vandana	0.089	0.805	0.074	0.033
WAGWAG	0.201	0.631	0.009	0.16
ZHENSHAN2	0.02	0.097	0.017	0.866
